# Effect of the Alkali-Sulphate Activators on the Hydration Process of Blast-Furnace Slag Mortars and Pastes

**DOI:** 10.3390/ma18030514

**Published:** 2025-01-23

**Authors:** Lei Li, Aveline Darquennes, Kinda Hannawi, Caigan Che

**Affiliations:** LGCGM, INSA Rennes, Université de Rennes, 20 Avenue des Buttes de Coësmes CS 70839 35 708 RENNES CEDEX 7, 35700 Rennes, France; kinda.hannawi@insa-rennes.fr (K.H.);

**Keywords:** activation energy, alkali activation, blast-furnace slag, hydration, setting, sulphate, thermodynamic modeling

## Abstract

The alkali-activation of blast-furnace slags (BFSs) is a topic largely studied today. However, some types of activators, more environmentally friendly, have been less studied such as alkali-sulphate activators. In this study, the effect of four alkali-sulphate activators (Na_2_SO_4_, K_2_SO_4_, MgSO_4_, CaSO_4_.2H_2_O) is investigated to better understand the effect of cations (Na^+^, K^+^, Mg^2+^, Ca^2+^) and of a high content of sulphate ions (SO_4_^2−^) on the hydration process of BFS and the nature of the hydrates. To reach this objective, a large experimental campaign is carried out to characterize the pore solution, the hydration products and the kinetics of the chemical reactions. As the temperature seriously affects the hydration advancement, the activation energy coefficient is also determined experimentally to compare the results as function of the equivalent time. Finally, a new method is proposed to determine the evolution of the hydration degree of BFSs, a key parameter for predicting the evolution of the hydrates through a thermodynamic modeling. The results indicate that the use of sodium sulphate results in faster hydration kinetics and shorter setting times due to a higher pH of their pore solution, leading to a larger rate of C-A-S-H type gel precipitation from the initial setting time to the long term and a higher hydration advancement. These hydration products are characterized by a higher content of Na^+^ and a denser rim around the surface of anhydrous particles. The effect of K_2_SO_4_, MgSO_4_ and CaSO_4_.2H_2_O on the BFS activation efficiency is limited compared to Na_2_SO_4_ due to their lower rate of C-S-H type gel evolution at early age. It is directly related to the pH of the pore solution and the effect of cations on the nature of hydrates. However, the compressive fis research study, a large strength beyond 28 days is more significant for mortars activated with Na_2_SO_4_ and MgSO_4_, satisfying the strength requirement of the repaired mortars (R2 and R3) due to the larger contents of C-(N)-A-S-H/M-S-H-type gels, ettringite and hydrotalcite.

## 1. Introduction

For decades, binders with a high content of mineral additions—often named eco-materials—have been extensively used to partially reduce the content of classic Portland cement (PC) and its environmental impact. Indeed, the reduction in the use of clinker, the main compound of PC, leads to a decrease in the carbon footprint of concrete [1]. One of the typical and commonly used mineral additions is blast-furnace slag (BFS) [1,2,3,4,5,6], as it allows improving the durability properties of concrete [4,5,6]. BFS is a by-product of the metallurgical industry used in Europe to produce blended cements such as CEM II and CEM III [4,7,8,9]. To achieve an appropriate hydration kinetics and a favorable strength, BFS can be activated by several methodologies to accelerate its chemical reactions, such as mechanical grinding [10,11,12,13], thermal treatment [14,15,16,17,18], and chemical activation [1,3,19,20,21,22,23,24,25,26,27,28,29]. A large part of the studies on BFS activation focuses on this last approach with the use of chemical alkaline activators such as sodium silicate (Na_2_SiO_3_), sodium hydroxide (NaOH), sodium carbonate (Na_2_CO_3_), and sodium sulfate (Na_2_SO_4_). Today, NaOH and Na_2_SiO_3_ are the most used and studied activators, followed by Na_2_CO_3_, Na_2_SO_4_ [1,3,19,20,30,31,32,33,34]. Because the alkali silicates and hydroxides lead to a faster hydration kinetics of BFS [3,19,31,33] and to significant mechanical strength [1,3,20,31,32,35,36], sometimes their strength levels are superior to those observed for Portland cement. However, these activators present some limits, such as a rapid setting [19,33] and significant cost [4,9,34]. Moreover, they are corrosive and not healthy [1,6]. In comparison with these activators, alkali carbonates and sulphates are more respectful to the environment [1,5,6], less expensive [1] and non-corrosive [37,38]. In particular, alkali-sulphate-activated BFS materials have gained enormous attention recently [26,27,29,39,40,41,42,43]. The autogenous shrinkage of alkali-sulphate-activated BFS is inferior to the activation of NaSiO_3_ and NaOH [30], but quite similar to that of PC [30,44].

Sodium sulphate also promotes faster early hydration than magnesium sulphate based on the Ca(OH)_2_ activation of BFS [45]. This indicates that the hydration behavior is significantly influenced by the specific cations associated with sulphate, as well as other cations released into the pore solution. However, while the effect of sodium on C-S-H-type gel formation, hydration processes, and the mechanical performance of sulphate-activated BFS has been more thoroughly studied [41,43,45,46], the effects of other cations, such as K⁺, Mg^2^⁺ and Ca^2^⁺, remain less well understood. Therefore, this study aims to investigate the influence of the four alternative sulphate activators characterized by different cations on the hydration process of BFS through a large experimental campaign and thermodynamic modeling. These activators are sodium sulphate (Na2SO4), potassium sulphate (K2SO4), magnesium sulphate (MgSO4) and dihydrated calcium sulphate (CaSO4.2H2O). The main objective is to determine the effect of the nature of the cation and a high content of sulphate ions (SO_4_^2−^) on the hydration process and the nature of the hydrates as well as on the compressive strength.

## 2. Materials and Methods

The effect of four alkali activators on the hydration process of mortars characterized by a high content of blast-furnace slag (BFS) is studied: sodium sulphate (Na_2_SO_4_), potassium sulphate (K_2_SO_4_), magnesium sulphate (MgSO_4_) and dihydrated calcium sulphate (CaSO_4_.2H_2_O). Their main characteristics are given in Table 1. Mortars activated with these products are hereafter named NS, KS, MS and CS, respectively. Moreover, a minor content of Portland cement (PC) CEMI 52.5 N is added to accelerate the setting. Tap water and natural sand 0/4 mm are used. The absorption coefficient of sand is equal to 0.8%. The chemical compositions of BFS and PC are presented in Table 2. BFS presents a larger content of particles inferior to 10 μm in comparison to PC. Indeed, their *D*_50_ determined by laser granulometric in ethanol solution is equal to 8.9 μm and 13.44 μm, respectively, and their specific surface area determined by gas sorption analysis to 1.03 m^2^/g and 1.35 m^2^/g.

The mixture proportions of NS, KS, MS and CS are given in Table 3. The binder (B) contains 86% BFS, 5% PC. The alkali-sulphate activator content of NS—the reference mortar in this study—is 8% of binder weight. The studied mortars are characterized by the same paste volume (Table 3), and each activator presents the same sulphate ions concentration (Table 3). The water-to-binder ratio (W/B) is similar for NS, KS and MS, but slightly higher for CS to ensure a slump value similar to that of MS (Table 3). Based on several literature studies [15,27,40,41,46] on Na_2_SO_4_-activated BFS pastes and mortars, the concentration of equivalent Na_2_O for NS is limited to 4% (% taken on mineral additions’ weight) to ensure a satisfied compressive strength and to limit the efflorescence risk. To ensure a homogeneous distribution of the activators during mixing, the Na_2_SO_4_, K_2_SO_4_ and MgSO_4_ activators were previously totally or partially dissolved into the added water as a function of their solubility (Table 1 and Table 3), except for the CaSO_4_.2H_2_O characterized by a low solubility. In the latter, its solid particles are directly mixed with PC and BFS. As the dissolution of MgSO_4_ is strongly exothermic, an ice-water bath dissolution method [47] is applied to limit the temperature increase and to obtain a solution at 20 °C. The mixing procedure is similar for all the studied mortars: (1) manually mixing the solid dry particles (BFS, PC, sand, activator), (2) adding the liquid solution (water with dissolved activator) and mixing with a slow velocity during 2 min, (3) mixing all the mixture with a rapid velocity during 2 min. The temperature at the end of the mixing process is close to 20 ± 1 °C for all the studied mortars.

A large experimental campaign is carried out to characterize the pore solution, the hydration kinetics and hydrates, as well as the mechanical performances. After mixing, all specimens are stored in a humidity chamber at 20 ± 1 °C and 95 ± 2% of Relative Humidity (RH). They are demoulded at 2 days (except MS demoulded at about 4 days) and maintained under autogenous conditions thanks to a layer of plastic foil and a glued double-layer of the aluminium sheet at 20 ± 1 °C and 50 ± 10% RH.

The pH and alkali ion concentration into the pore solution are two parameters affecting the hydration kinetics, particularly at early age. The pH of the pore solutions for each studied mixture is measured at 0, 3, 7, 28, 90 and 365 days using a glass pH electrode with a precision of 0.01 on three specimens from the same mixture. For the hardened mortars, specimens are taken from the centre of cylindrical mortars (diameter Φ× height h: 4 × 6 cm) and crushed to obtain a powder with a particle size inferior to 315 μm. A total of 1 g of the powder is mixed with 10 mL of distilled water as recommended in [39]. The solution is stirred magnetically for 24 h and then filtered using 0.7 µm filtered papers. Moreover, the measurement of alkali ions (Na^+^, K^+^, Mg^2+^ and Ca^2+^) is also performed at 3, 7, 28, 90 and 365 days. In this case, 1 g of powder is mixed with 150 mL of distilled water and the ion concentration is measured using ion chromatography (Dionex DX-100, Sunnyvale, CA, USA) after 24 h of agitation. Three measurements of the concentration are made on the same solution with an accuracy of 0.001 ppm.

The hydration kinetics at early age is studied by means of an isothermal calorimeter (Calmetrix I-CAL 2000, Arlington, MA, USA) by measuring the heat release at 10, 20 and 30 °C. Based on these measurements, the cumulative heat release *Q*, a hydration advancement degree $\alpha_{th}$ and the activation energy *E_a_* are also quantified. One day before the test, all the material is placed in the calorimeter to obtain a mortar temperature after manually mixing close to the test temperature. The mixing time is equal to about 2 min and the tested mortar volume is equal to 59 cm^3^. The duration of the test performed in the same time on two samples is 96 h.

To determine the hydration advancement degree $\alpha_{th}$ (Εquation (1)) calculated with the heat release due to the chemical reactions, the cumulated heat evolution *Q*(*t*) at 20 °C is predicted with the Three Parameter Model (CPM) proposed by Pane and Hansen [48] using Equation (2) and the cumulated heat evolution *Q*(*t*) determined from the heat flow curve initialized at its first peak. The two parameters $\tau_{th}$ and $a_{th}$, controlling the intercept and curvature of the plot in the logarithmic scale, respectively, are calibrated using the Nonlinear Least-Square Fitting method. The ultimate heat value $Q_{\infty ,20}$ is determined using a graphical extrapolation method [48,49] in which the cumulated heat evolution is plotted as function of $\frac{1}{t}$ and extrapolated by a linear function when $\frac{1}{t}$ tends to 0.

(1) $$\alpha_{th}\left(t\right)=\frac{Q\left(t\right)}{Q_{\infty ,20}}$$

(2) $$Q\left(t\right)=Q_{\infty ,20}\exp\left[-{\left(\frac{\tau_{th}}{t}\right)}^{a_{th}}\right]$$

To take into account the temperature effect on the hydration kinetics, the activation energy *E_a_* is calculated using two methods: superposition and velocity methods. The superposition method is based on the Arrhenius law [49] and the equivalent time *t_eq_* (Equation (3)). The two cumulated heat curves obtained at 10 and 30 °C are overlayed as a function of the equivalent time and their deviations are minimized using the Nonlinear Least-Square Fitting method on the cumulative heat interval [*Q_inf_*, *Q_sup_*] determined with Equations (4) and (5). From this method, a constant value is obtained for the activation energy. On the contrary, the velocity method offers the evolution of *E_a_* as a function of the hydration advancement degree $\alpha_{th}$. This calculation (Equation (6)) is based on the instantaneous rates of hydration for a given degree of hydration advancement using the cumulated heat release curves at 10 and 30 °C [49].

(3) $$t_{eq}\left(h\right)=\sum \left\{exp\left[-\frac{E_{a}}{R}\left(\frac{1}{T_{i}}-\frac{1}{T_{r}}\right)\right]\cdot ∆t\right\}$$

(4) $$\begin{array}{c} Q_{inf}={\bar{Q}}_{\infty }\cdot \left(0.16+0.88\frac{{Rc}_{inf}}{{Rc}_{28}}\right) \end{array}$$

(5) $$Q_{sup}={\bar{Q}}_{\infty }\cdot \left(0.16+0.88\frac{{Rc}_{sup}}{{Rc}_{28}}\right)$$

(6) $$E_{a}(J/mol)=-\frac{R}{\frac{1}{T_{1}\left(Q\right)}-\frac{1}{T_{2}\left(Q\right)}}ln\left(\frac{{\left(\frac{dQ}{dt}\right)}_{1}\left(Q\right)}{{\left(\frac{dQ}{dt}\right)}_{2}\left(Q\right)}\right)$$

where R is the universal gas constant equal to 8.314 J/(mol.K), *T_i_* (K) is the average temperature in the time interval Δ*t, T_r_* (K) is the reference temperature at 20 °C, ${\bar{Q}}_{\left(\infty \right)}\;$is the average value of cumulative heat ad infinitum, *Q_inf_* and *Q_sup_* are the lowest and highest heat values in the cumulative heat interval, *Rc*_28_ is the 28-day compressive strength at 20 °C, *Rc_inf_* is a compressive strength equal to 0 MPa, *Rc_sup_* is a compressive strength equal to *Rc*_28_/2, *T*_1_ and *T*_2_ temperatures equal to 10 and 30 °C, respectively, and ${\left(\frac{dQ}{dt}\right)}_{i}$ is the heat flow related to the fixed heat (*Q* = *Q*_0_, *Q*_1_,…).

Thermogravimetric Analysis (TGA) is used to monitor the hydration process at early age (<3 days) and at long term (3, 7 28, 90 and 365 days). The test is carried out on two samples for each mixture. The tested specimens are obtained from the centre of mortar samples (4 × 4 × 16 cm) and dried at 45 °C for 24 h in a vacuum oven to avoid the carbonation. After, they are ground to obtain a powder characterized by a particle size inferior to 80 µm. The powder (around 60 mg) is placed in a nitrogen atmosphere and the temperature varies from 20 to 1000 °C with a heating rate of 10 °C/min. Notice that the sample is maintained at 105 °C for 20 min to ensure the evaporation of all the free liquid water. The mass loss evolution as a function of temperature is recorded and the mass loss content ∆*m_i_* is calculated with Equation (7).

(7) $${\Delta m}_{i}\left(\%\right)=\frac{\left|{\delta m}_{i}\right|}{m_{initial}}\times 100$$

where *m_initial_* is the initial mass of the sample measured at 105 °C (g) and δ*m****_i_*** is the mass variation on the temperature interval *i* (g).

Three main temperature ranges are chosen to consider the mass variation related to the decomposition of hydration products:

(1) [105–250 °C] related to the first peak and mainly to the decomposition of C-A-S-H-type gel [8,24,50,51] but also to the decomposition of M-S-H-type gel for MS [47,52];
(2) [300–400 °C] linked to the second peak and to hydrotalcite and/or brucite decomposition [23,24,27];
(3) [105–500 °C] corresponding to the decomposition of chemical-bound water [8,24,53].

A hydration advancement degree $\alpha_{TG}$ is evaluated using the mass loss related to C-A-S-H/M-S-H-type gels located in the interval of [105–250 °C] [48,54], as well as Equations (8) and (9) based on the CPM model. The ultimate value of the mass loss related to C-A-S-H/M-S-H-type gels (*χ_∞_*) as well as parameters $\tau_{TG}$ and $a_{TG}$ are calibrated using a Nonlinear Least-Square Fitting method [48,54].

(8) $$\chi \left(t\right)=\chi_{\infty }\exp\left[-{\left(\frac{\tau_{TG}}{t}\right)}^{a_{TG}}\right]$$

(9) $$\alpha_{TG}\left(t\right)=\frac{\chi (t)}{\chi_{\infty }}$$

Setting time is determined thanks to the velocity of compressive waves moving through a cylindrical mortar sample (Φ: 8 cm, h: 4 cm). Its propagation is monitored using two ultrasonic transducers with a frequency of 54 kHz (Vikasonic device, Schleibinger Geräte Teubert u. Greim GmbH, Buchbach, Germany). The test parameters are a pulse rate of 1 s and a voltage of 2000 V. The measurement begins directly after mixing and is recorded for 4 days. The specimens were are at 20 ± 1 °C. The mortar temperature is also monitored thanks to a thermocouple. From the measurements, the curve of velocity *V* as function of time *t* is fitted for each studied mixtures with a typical curve using the multi-logistic model (Equation (10)) [19,55]. For this model, the four parameters (with *i* = 1; 2; 3) are calibrated using the Nonlinear Least-Squares Fitting method: the asymptotic parameter (*V*_i_), the time characteristics corresponding to the inflexion points (*t_i_*), the slope parameter (*g_i_*) and the baseline value (*C*).

(10) $$V\left(t\right)=\sum_{i}\frac{V_{i}}{1+e^{\frac{t-t_{i}}{g_{i}}}}+C$$

Three characteristic points are determined on the velocity curve: two inflection points corresponding to the moment when the second derivative of the velocity curve is equal to zero named the initial setting time (IST) and the final setting time (FST) in agreement with previous research works on Portland cement and BFS blended cement [55], and the Plateau time (PT) is defined as the second minimum of curve corresponding to the second derivative. This methodology to determine IST is also validated on a geopolymer [56] and alkali-activated BFS/FA binders [57]. But this approach has to be investigated further to determine FST on materials with a high content of mineral additions as discussed in the present study.

XRD analysis and SEM observations are performed on 28-day-old paste samples for each mixture. Their formulations are based on the mortar proportions (Table 3) in which the water content is slightly reduced to take into account the water adsorbed by the sand. Their water-to-binder ratio is slightly modified and is equal to 0.4 for NS, KS and MS, and 0.48 for CS. For the XRD measurements, the powder located in the centre of the samples (4 × 4 × 16 cm) is ground to a diameter inferior to 80 µm. Measurements are performed from 5 to 80 °(2θ) with a step size of 0.02° on specimens turning with a speed of 10 t/min. The power generator (Cu Kα1) is operated at 30 kV and 10 mA. The content of the crystalline phases is determined with the software EVA (https://www.bruker.com/en/products-and-solutions/diffractometers-and-x-ray-microscopes/x-ray-diffractometers/diffrac-suite-software/diffrac-eva.html, accessed on 29 October 2024). The SEM observations are performed on paste slices impregnated with epoxy resin and subjected to successive polishing (1200 µm, 9 µm, 3 µm and 1 µm) for approximately 4 h [58]. The SEM system is configured to acquire at least four BackScatter Electron (BSE) images for each mixture, and also to perform Energy Dispersive Spectrometer (EDS) element analysis on several points of images. An accelerating voltage of 20 kV and a working distance of approximately 10 mm are utilized for SEM-BSE/EDS. For EDS, the pointing analysis with 5~10 measurements on the hydrated products is conducted on each picture. In order to clearly observe the anhydrous and hydrated particles in a 2D surface, a high magnification of 1:1000 or 1:3000 is selected on at least three typical images for each studied mixture. The SEM-BSE pictures are segmented to determine the anhydrous BFS volume fraction at 28 days using a K-means clustering method [58,59]. The segmentation method is an algorithm for grouping data points from each image pixel into clusters, iteratively adjusting the cluster centers in space location until there is no significant change.

Thermodynamic modeling is used to better understand the influence of the activator type on the hydrates formed. It is carried out using Gibbs free energy minimization program GEM-Selektor v.3.7 (http://gems.web.psi.ch/) [60,61]. The GEM (Gibbs free energy minimization) approach is based on a mass and charge balance of the whole system, and the equilibrium composition is found from all the stoichiometrically possible phase combinations. From the mass action constant *K* at different temperatures, the Gibbs free energy of reaction ${∆}_{r}G^{o}$ (kJ/mol) and the Gibbs free energy of formation for each individual compound ${∆}_{f}G^{o}$ (kJ/mol) are calculated with Equation (11),

(11) $${∆}_{r}G^{o}=\sum_{i}v_{i}{∆}_{f}G^{o}=-RTlnK$$

where $v_{i}$(-) is the stoichiometric coefficients of the reaction, *R* is the universal gas constant (8.31451 J/mol/K) and *T* (K) is the temperature.

The Gibbs free energy minimization approach has the advantage of no a priori assumptions having to be made about the phases present, the compositions of solid solutions, pH, redox potential and the fugacity of gases; these parameters are obtained as output parameters [62]. Thus, GEMs can compute an equilibrium phase assemblage and speciation in a complex chemical system with many phase solutions from its total bulk elemental composition at given temperature and pressure [63].

The software is coupled with the PSI-GEMS database containing thermodynamic data for aqueous species as well as for many solids [64] and the CEMDATA18 database [62] allowing to calculate the evolution of the hydration products of alkali-activated binders. Indeed, this thermodynamic database contains models for the calcium (alkali) aluminosilicate hydrate C-(N)-A-S-H gel [65,66] with a lower calcium but higher aluminum and alkali content than in the C-S-H-type phase which exists in hydrated Portland cement. The thermodynamic characteristics of this gel used in the modeling are given in [65,66]. The product MA-OH-LDH [67] with variable Mg/Al ratio is also available [62,66], as well as the M-S-H gel [68]. For the present numerical simulations, the reaction of the Portland cement is modeled using the empirical approach of Parrot and Killoh [69], and the evolution of the hydration degree used for BFS is determined from SEM-BSE and TGA experimentations presented in Section 3.4. The input parameters for the thermodynamic model are the chemical compositions of BFS and PC (Table 2), the water and binder content (Table 3) and the curing conditions. The content of the hydration products is determined at 20 °C for a hydration degree of BFS equal to 0.01, 0,02, 0.10, 0.20, 0.30, 0.40, 0.50, 0.60 and for the final hydration degree of BFS.

Compressive strength is performed on prismatic specimens (4 × 4 × 16 cm) according to NF EN 196-1 [70,71] at 3, 7, 28, 90 and 365 days on three specimens for each age. The loading velocity is equal to 2.4 kN/s.

The pore solution (pH and alkali cations concentration) is first characterized to analyze the effect of the studied activators on the dissolution of BFS. Then, the hydration process at early age (<3 days) is monitored by coupling several parameters: the heat flow of the hydration process, the formation of hydrates and the setting. To compare these results, it is necessary to take into account the temperature effect on the hydration process. So, the activation energy is determined using two methods. For the long term, the hydration products are also analyzed thanks to several experimental techniques (TGA, XRD, SEM) at a paste scale. Based on these experimental results, a new approach is proposed to determine the hydration degree of blast-furnace slag activated with alkali-sulphates. It is valorized through two applications: the prediction of the hydration products using a thermodynamic modeling and its correlation with the evolution of compressive strength.

## 3. Results and Discussion

### 3.1. Properties in the Fresh State

Table 3 shows the average values of slump and air content in the fresh state determined from three tests. The average standard deviation is equal to 3.2 mm and 0.1%, respectively. The last parameter using an aerometer is quite similar for all studied mortars. But the rheological behavior of the mixtures is significantly affected by the activator type. The NS mortars exhibit the best workability with a slump value equal to 26 mm. It can be related to the two main reasons as presented in Table 1: (1) superior solubility and (2) higher alkalinity as shown by its pH. A higher solubility for NS (Table 1) leads to the more anionic exchange with 2 Na molecular released into the solution and high conductivity of the activating solution, promoting the dissolution rate of anhydrous particles and its dispersion performance. The higher pH of the activator solution also favors the slag dissolution due to the increase in the concentration of hydroxyl (OH^−^) [72]. On the other hand, the slump value is reduced with the other activators, particularly for MS and CS (6 mm—Table 3). It can be related to its lower pH (Table 1). In addition, MgSO_4_ presents a endothermic reaction, promoting more dissolution of activator to absorb the heat energy partially [73] instead of the BFS dissolution. CaSO_4_.2H_2_O shows higher water absorption capacity, limiting the availability of free water needed for lubrication and effective particle dispersion [74] as characterized by a monocline structure [75].

### 3.2. Pore Solution Chemistry

**Figure 1:**
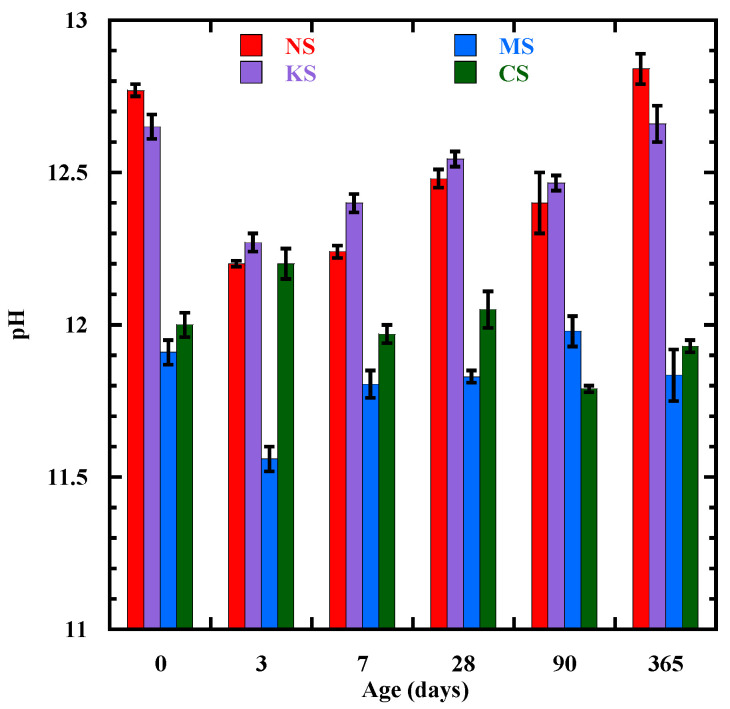
The evolution of pH in the pore solution from the end of the mixing (time “0”) to 365 days.

Figure 1 presents the pH evolution of the studied BFS mortars from 0 to 365 days. The initial pH of the pore solution just after mixing (time “0”) is the highest for NS (12.77) and KS (12.65) (Figure 1). Its value is inferior to 12 for MS and CS. It is mainly related to the type of activators, as shown in their pH measured in distilled water (Table 1). Indeed, this pH is superior to activators Na_2_SO_4_ and K_2_SO_4_ (10) as well as BFS only in the distilled water (9.1). At 3 days, a pH superior to 12 is observed for all the studied mortars, except for MS (11.5) (Figure 1). A pH below 12 for MgSO_4_-activated slag samples is also observed at 3 days by Kang et al. [76]. For NS and KS, the drop in pH from the day after mixing to 3 days is more important due to a stronger hydrolysis effect of Na^+^ and K^+^ [46,77,78] in comparison to that of Mg^2+^ and Ca^2+^. Beyond 3 days, the pH of NS and KS is increasing and reaches a value equal to 12.8 and 12.7 at 365 days, respectively. These values are in agreement with the previous study carried out by Rashad et al. [27]. They find that the pH of BFS activated with 1~3% Na_2_SO_4_ varies from 12.2 to 12.5 for three first months. In their case, they observe a decrease in the pH during the first month and a stabilization from 60 to 90 days. The fluctuation of pH values could be attributed to the chemical reaction with the potential presence of Ca(OH) [45]. The increase in pH is quite lower for MS and its value at 1 year old is equal to 11.8. On the other hand, the pH of CS decreases from 3 to 365 days to reach a value equal to 11.9.

**Figure 2:**
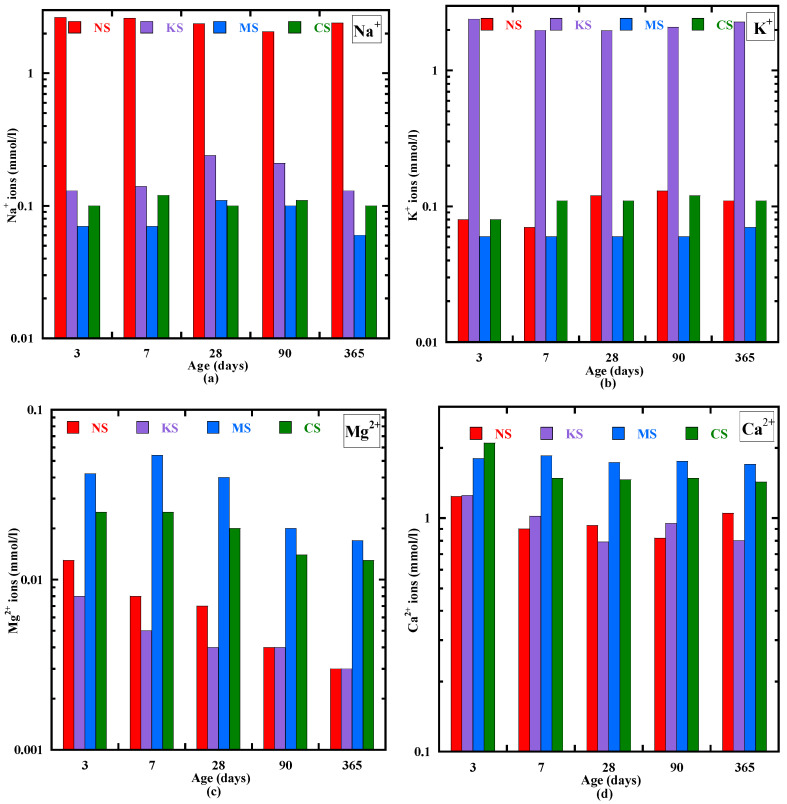
Concentration of Na^+^ (**a**), K^+^ (**b**), Mg^2+^ (**c**) and Ca^2+^ (**d**) in the pore solution from 3 to 365 days.

Figure 2 presents the evolution of the Na^+^, K^+^, Mg^2+^ and Ca^2+^ ion concentrations into the pore solution from 3 to 365 days. The initial concentration of alkali ions for each mixture after mixing is neglectable (close to zero). From 3 to 365 days, the concentration of all the ions is inferior to 3 mmol/l. The concentration of Ca^2+^ is the same order of magnitude for all the mixtures at 3 days (1–2 mmol/l) (Figure 2d). It is mainly due to the dissolution of the Portland cement, but the highest value is observed for CS, confirming the partial dissolution of activator CaSO_4_.2H_2_O. A significant decrease in the concentration of Ca^2+^ is observed from 3 to 7 days for NS, KS and CS. Its value stays stable for MS. Beyond 7 days, the concentration of Ca^2+^ decreases weakly for all the studied mixtures, but KS presents a slightly higher decrease rate (0.4%). The concentrations of Na^+^, K^+^ and Mg^2+^ are the highest in the mixtures activated with Na_2_SO_4_, K_2_SO_4_ and MgSO_4_, respectively (Figure 2). At 3 days, they are equal to 2.6 mmol/L, 2.4 mol/L and 0.04 mol/L, respectively. So, the highest pH value for NS and KS (Figure 1) is due to the highest content of Na^+^ and K^+^ in their pore solution. This observation is in agreement with the study of Huang et al. [46] on the NaOH/KOH-activated BFS binders. The Na^+^ concentrations is basically constant and it is equal to 2.3 mol/L at 365 days. A small decrease in this ion content in the pore solution is possibly related to their consumption to produce new hydration products during the curing time. For the Na^+^ concentration, a different behavior is observed for MS, CS and more particularly for KS: it slightly increases during the hydration process, then decreases to reach a value at 1 year close to the concentration at 3 days. Quite similar evolution is also observed for the K^+^ concentration in the pore solution of NS and CS, whereas it stays constant for MS. It appears that the evolution of Na^+^ and K^+^ concentration is strongly related to the C-(N)-A-S-H-type gel formation. However, the Mg^2+^ concentration decreases for all the studied mixtures during the first year, possibly related to the formation of brucite/M-S-H [45].

### 3.3. Hydration Process at Early Age

The effect of the alkali-sulphate activators is investigated on the hydration process (hydration kinetics, degree of hydration advancement, setting, activation energy, hydration products) from the fresh state to 3 days using several experimental technics such as the isothermal calorimetry, the thermogravimetry and the propagation of ultrasonic waves.

#### 3.3.1. Hydration Kinetics

**Figure 3:**
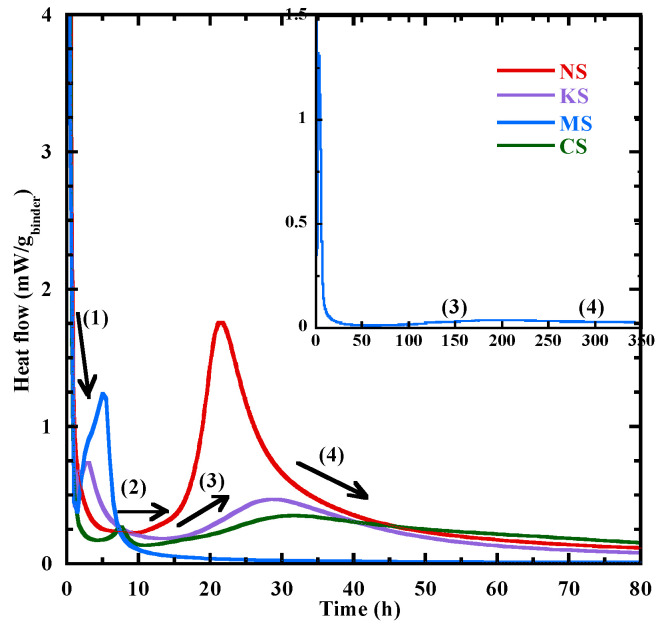
Heat flow evolution for all the studied mortars during the first 3 days.

The heat flow at 20 °C during the first 3 days is shown in Figure 3 for all the studied mixtures. It can be divided into four periods [19,48,55]: (1) dissolution period; (2) dormant period; (3) acceleration period; (4) deceleration period. The main characteristic times are given in Table 4. To relate them to the formation of hydrates, TGA is performed at the same time on specimens whose hydration process is stopped following the method proposed in [79]. The average mass loss related to the decomposition of hydration products is presented in Table 5. Its standard variation is inferior to 0.05%.

After the sharp initial peak related to the dissolution period, NS presents a dormant period with a flat heat flow before the acceleration period. This behavior is close to that observed for the Portland cement. A different behavior characterizes the hydration kinetics of other mortars. Their first peak is rapidly followed by a second peak. This last occurs at 3.1 h, 3.3 h and 7.6 h for KS, MS and CS, respectively (Table 4). These peaks are mainly linked to the formation of portlandite and brucite/hydrotalcite [20], but Table 5 shows a low content of these products, as the mass losses at the second peak is inferior to 0.1%. These observations are confirmed by the thermodynamic modeling presented in Section 3.6.1. Furthermore, the effect of ettringite on the hydration kinetics cannot be ignored. A high content of sulphate on the PC leads to greater formation of ettringite and a morphological change in ettringite from spherical or stubby rod to elongated ettringite [80]. As a result, the yield stress and viscosity increase [80], leading to a higher slump value (Table 3) and a faster reaction for NS. The dormant period of these mixtures (KS, MS and CS) is defined as the time between the end of the second peak and the beginning of the acceleration period (b.a.p). It is the longest for MS (31 h) and the shortest for CS (2.5 h).

**Figure 4:**
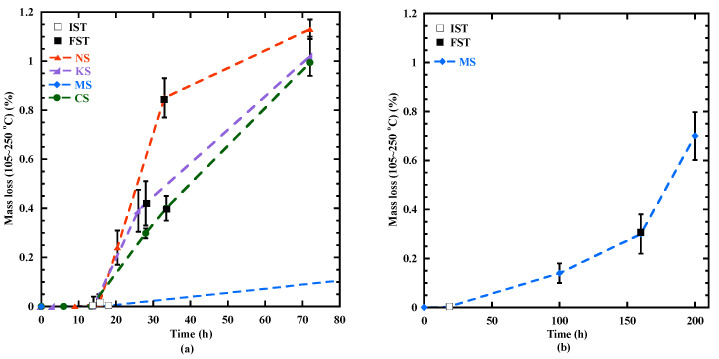
Evolution of mass loss (105–250 °C) related to the content of C-(N)-A-S-H- and M-S-H-type gel for all the studied mortars during the first 3 days (**a**) and for MS mortar up to 200 h (**b**).

**Figure 5:**
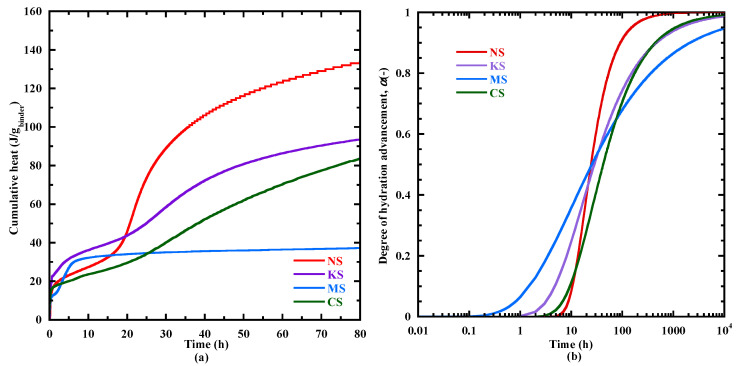
The evolution of the cumulative heat (**a**) and the hydration advancement degree *α_th_* predicted with the CPM model (**b**) for all the studied mortars.

$Q_{\infty ,20}$ At the beginning of the acceleration period, a rapid rise in the heat flow is observed. The mass loss related to the chemically bound water varies from 0.1 to 0.3% (Table 5) and the content of C-(N)-A-S-H type gel is negligeable, except for MS (0.3%) due to the formation of M-S-H gel at very early age (Section 3.6.1). During the acceleration period, the content of C-(N)-A-S-H/M-S-H-type gels increases significantly (Figure 4) and its mass loss at *the third peak* for NS, KS, CS and MS is equal to 0.24%, 0.38%, 0.29%, 0.5% respectively. Their hydration advancement degree $\alpha_{th}$ determined from the cumulative heat curves (Figure 5a) and the CPM model (Equation (2)) at *the third peak* is equal to 0.45, 0.5, 0.42 and 0.75, respectively. The ranking of hydration advancement degree at the thirrd peak: MS > KS > NS > CS. During this period, NS presents the fastest kinetics of C-(N)-A-S-H-type gel formation (4.7‰/h) (Figure 4a and Table 6) and of the hydration advancement degree $\alpha_{th}$ (3.8) (Figure 5b and Table 6). The contents of hydrotalcite, brucite and portlandite also increase (Table 5).

The deceleration period (from *the third peak* to 72 h) with a gradual decline presents a continuous increase in the amount of hydrates. At 72 h, NS, KS and CS have a close content of chemical-bound water and gel with a mass loss equal to about 2.3% and 1%, respectively (Table 5). $\alpha_{th}\;$at 72 h shows the following rank: NS (0.86) > KS (0.69) > MS = CS (0.64). The faster increases in the hydration advancement degree ($\alpha_{th}$) for NS are due to faster kinetics of C-(N)-A-S-H-type gel formation.

Based on these results, it appears that the sodium sulphate leads to an effective activation on the BFS hydration at early age, as shown by the reduction in the different characteristic times (Table 4) and a faster rate of hydration advancement degree $\alpha_{th}$ beyond the beginning of the acceleration period (Figure 5b) due to a faster formation of C-(N)-A-S-H (Table 6). It is related to the high pH of its pore solution at the end of mixing (Table 6—Figure 1). Although a similar pH value is observed after mixing and at 3 days for the pore solution of KS (Table 6—Figure 1), the potassium sulphate activation is less effective compared to sodium sulphate as shown by the time associated with the beginning of the acceleration period (Table 4), the kinetics of C-(N)-A-S-H formation (Figure 4) and of $\alpha_{th}$ on the acceleration period (Table 6—Figure 5b). This difference of behavior is directly related to the cation nature of the activator. Indeed, Na^+^ ions are characterized by a higher ionic polarization with the dissolved anions in the pore solution [46,81], and the binding of Na^+^ ions into C-(N)-A-S-H-type gels is linearly proportional to its concentrations in the pore solution, resulting in accelerated hydration [41,46]. Conversely, K^+^ ions prefer to bind with outer products than the inner products of the C-A-S-H in the alkali-activated BFS [51,81,82,83], leading to reduced formation of C-A-S-H-type gel and a slower hydration process as shown in Figure 4a. The use of CaSO_4_.H_2_O and MgSO_4_ leads to a lower heat release (Figure 5a) and slower hydration kinetics at early age (Figure 4) due to the lower pH in their pore solution (Figure 1—Table 6). The study of Fu et al. [12] shows that a lower pH slows down the hydrolysis of the Si-O bond [12], leading to a reduction in the dissolution rate of BFS anhydrous particles.

#### 3.3.2. Temperature Effect on the Hydration Process

**Figure 6:**
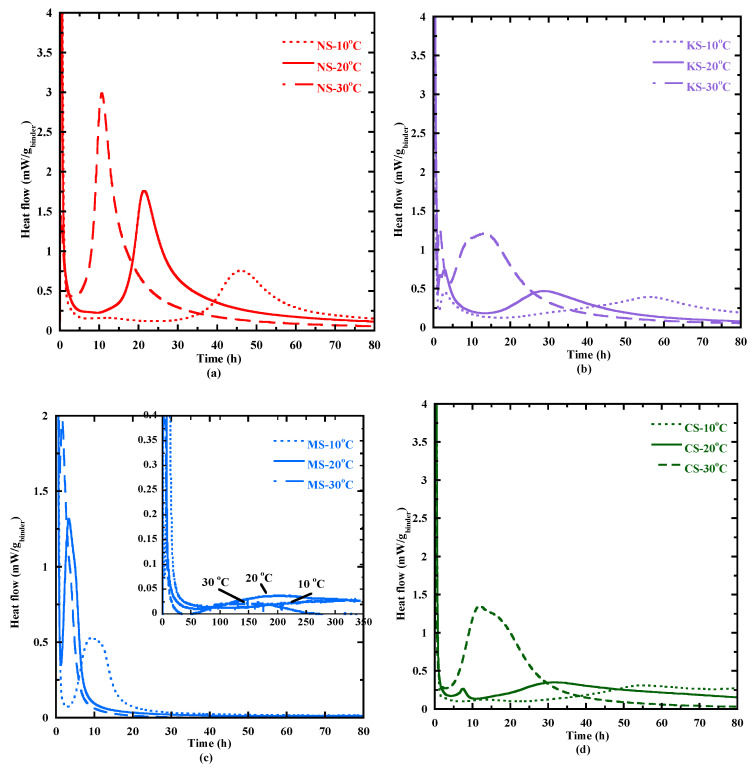
Heat flow curves at 10, 20 and 30 °C for NS (**a**), KS (**b**), MS (**c**) and CS (**d**).

The effect of curing temperature (10 °C, 20 °C and 30 °C) on the heat flow is presented in Figure 6. Globally, the temperature effect is the same for all the studied mixtures: a temperature reduction at 10 °C leads to an increase in the different characteristic times, whereas temperature increases at 30 °C lead to a reduction in these times (Figure 6—Appendix A). For example, the dormant period of NS decreases from 20.8 h to 2.4 h for a curing temperature varying from 10 to 30 °C, respectively. The shape of heat flow at 10 and 30 °C is quite similar to that at 20 °C for NS, KS and MS. These two last mortars present three peaks for all the studied temperatures, but their appearance time increases at 10 °C and reduces at 30 °C. For CS, the second peak occurs right before the third peak, leading to a double peak between 5 and 30 h at 30 °C. It is possibly attributed to the transformation of ettringite into monosulfoaluminate as observed for Portland based-cement [84,85]. From these results, it appears that a temperature curing at 30 °C is beneficial to accelerate the hydration process at early age. To better take into account this effect, the activation energy coefficient is determined to quantify the temperature sensitivity of the hydration process for all the studied mortars using two methods: the velocity and superposition methods.

**Figure 7:**
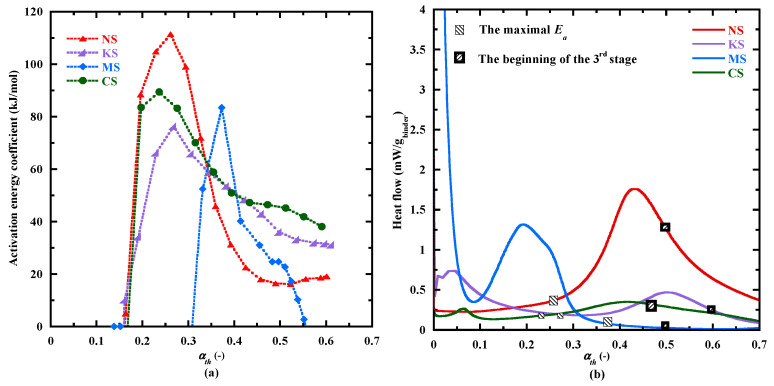
Evolution of activation energy coefficient determined with the velocity method as function of the hydration degree advancement *α_th_* (**a**) and the heat flow curves measured at 20 °C as function of *α_th_* (**b**).

Figure 7a displays the evolution of activation energy coefficient *E_a_* calculated with the velocity method (Equation (6)) as a function of the hydration advancement degree $\alpha_{th}$. It presents four stages:

- The first stage is characterized by a fast increase in *E_a_*. It begins at a $\alpha_{th}$ equal to about 0.16 for NS, KS and CS and to a higher value for MS (0.31) (Table 7). The highest value of *E_a_* (111 kJ/mol—Table 7) is reached by NS at $\alpha_{th}$ equal to 0.26. For NS, KS and CS, the maximal *E_a_* values are reached near the end of the dormant period, indicating a similar kinetic behavior during the early hydration phase (Figure 7b). Indeed, under a similar $\alpha_{th}$ (0.25), *E_a_* is equal to 76 kJ/mol for KS and 89 kJ/mol for CS. For MS, the slightly delayed peak could be attributed to its slow reaction kinetics (Figure 3), affecting its dormancy duration (Figure 7b).
- During the second stage, *E_a_* decreases until it reaches a quite stationary state (beginning of the third stage). This stage happens during the acceleration period for NS, KS and CS, highlighting the important effect of physical and chemical changes during this stage (Figure 7b). The drop in *E_a_* confirms an increased ease of reaction and a stable establishment of hydration products.
- The third stage begins at an $\alpha_{th}$ equal to about 0.46 for CS, 0.49 for NS, 0.5 for MS and 0.6 for KS. The average value of *E_a_* calculated on this stage is equal to 46, 18, 24 and 32 kJ/mol, respectively. In this stage, the hydration process of CS and KS are more sensitive to thermal activation.
- For CS and MS, a second decrease is observed (fourth stage), possibly related to their larger duration of the characteristic times (Appendix A) and their lower evolution rate of heat flow at 10 °C (Figure 6). This secondary reduction could be associated with the prolonged hydration and diminished thermal sensitivity [86].

Based on the velocity method, an average activation energy (*E*_a,*VM*_) is determined on a cumulative heat interval [*Q_inf_*, *Q_sup_*], corresponding to the $\alpha_{th}$ interval equal to [0.16–0.6] for NS, KS and CS and equal to [0.3–0.55] for MS (Table 7). These values of *E*_a,*VM*_ are superior to that obtained on the third stage, likely due to the its sensitivity to instantaneous reaction rate [87]. In comparison, the superposition method produces a constant activation energy value (*E*_a,SM_) over the same interval (Table 7). Although *E*_a,*VM*_ is slightly higher, the deviation between the two methods is minimal when considering the standard variation of 5 kJ/mol [7,49], except for CS, where the difference is more pronounced. This may indicate that the velocity method captures more fluctuations for CS hydration. In addition, MS is less sensitive to the thermal activation than NS, KS and CS. The values of *E*_a,SM_ [33–40 kJ/mol] are in the same range as those determined for CEM Ⅲ-type binders [30–40 kJ/mol] [7,18,49], but lower than those of silicate sodium-activated BFS binders [50–55 kJ/mol] [18]. Finally, *E*_a,SM_ is chosen to take into account the effect of the temperature during the setting thanks to the equivalent time (Equation (3)). These results are presented in Section 3.3.2.

#### 3.3.3. Setting

**Figure 8:**
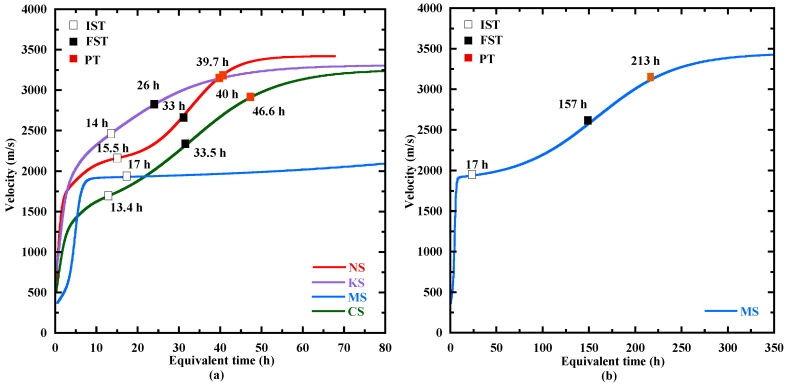
Evolution of the velocity for all the activated mortars during the first 3 days (**a**) and for MS until 350 h (**b**) with the initial setting time (IST), the final setting time (FST) and the Plateau time (PT).

The velocity evolution is presented as a function of the equivalent time in Figure 8. The velocity begins at a quite similar value for NS and KS (about 700 m/s). This value is inferior for CS (500 m/s) and MS (370 m/s). The initial velocity value for all the studied mortars is inferior to that of the dissolved activator solution for all the studied sulphate salts (about 1600 m/s), but superior to that in the air phase (340 m/s) [19,55]. These low values for the initial velocity are probably due to the interfaces between the different phases (solid, liquid and air) of the mortar at the fresh state. After, the velocity displays a rapid increase at early age, probably related to the ettringite formation as indicated by other authors [40,88,89]. Indeed, the sulphate salts favorize the formation of this product, which makes easier the travel of the compressive waves through the specimen. No XRD tests were performed to confirm this hypothesis as ettringite is difficult to be detected at very early hours [88,90] due to the effect of a high pH value in the activating solution on its crystallinity [19,85]. After, the kinetics of the velocity evolution slows down progressively until a value corresponding to the first point where the second derivative is equal to zero (Figure 8a). In agreement with previous studies [55,56,57,91], this point is defined as the initial setting time (IST). Beyond IST, the rate of velocity evolution increases until a point defined in the literature [22,24] as the final setting time (FST), corresponding to the second zero of the second derivative. This approach is discussed hereafter. Beyond FST, the evolution rate of velocity gradually decreases to tend to a constant value at a point named Plateau time (PT). The values of IST, FST and PT are given in Table 8.

**Figure 9:**
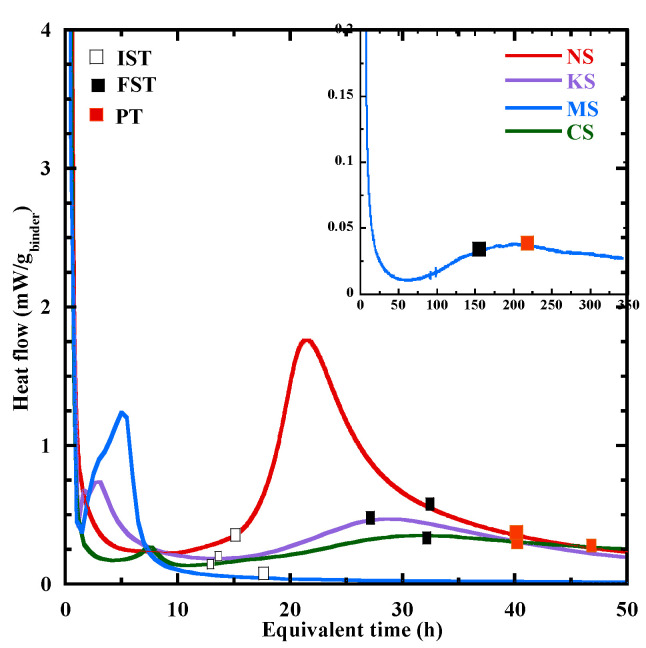
Heat flow curves with IST, FST and PT for all the studied mixtures.

The IST of CS (13.4 h), KS (14 h) and NS (15.5 h) are quite close. It appears later for MS (17 h). The IST is close to the end of the dormant period for all the studied mortars (Figure 9), except for MS. At this time, a higher velocity is observed for KS (2480 m/s) and NS (2187 m/s). It indicates that these matrixes are less porous and their ettringite content, a hydration product making easier the compressive waves transport, is probably more important. The FST is reached at 33 h for NS, 26 h for KS, 33.5 h for CS and 157 h for MS. The velocity at FST is again higher for KS (2879 m/s) and NS (2797 m/s) than for MS (2655 m/s) and CS (2331 m/s) (Table 8). For all the studied mixtures, the velocity increase from IST to FST is related to the formation of C-(N)-A-S-H ($∆m_{105-250{}^{\circ}C}$) as shown in Table 8, but the ettringite formation can also contribute to that. It results in reduction in porosity. But the position of FST on the heat flow curve differs as a function of the activation system, and it appears at different periods of the hydration process (Figure 3). It is located in the acceleration period for KS and MS and in the deceleration period for NS and CS (Figure 9). The choice of this point to define the final setting seems to be contestable. So, another characteristic point is analyzed: Plateau time (PT).

PT is quite similar for NS (39.7 h) and KS (40 h) as well as their corresponding velocity (3155 m/s and 3170 m/s, respectively). MS presents a similar velocity at its PT (213 h), whereas it decreases slightly for CS (2885 m/s). The PT for all the studied mixtures takes place during the deceleration period of the heat flow curves (Figure 9) and in the deceleration phase of the hydration advancement degree $\alpha_{th}\;$(Figure 5b). From FST to PT, the mass loss related to C-(N)-A-S-H- and M-S-H-type gels ($∆m_{105-250{}^{\circ}C}$) also increases significantly for MS (Table 8) as the acceleration period is not finished at FST. At PT, the mass loss related to C-(N)-A-S-H- and M-S-H-type gels varies from 0.5% to 1%. This content of hydrates coupled with the formation of ettringite allows the development of a matrix stiffness and the demolding of the specimens. Based on all this information, the choice of Plateau time is more relevant to define the end of the setting.

At PT, the velocity is quite similar for NS, KS and MS. The lower velocity for CS indicates that its matrix is more porous. For a same PT (around 40 h), the increase in velocity for NS and KS during the setting phase is more significant for NS. This is possibly due to its faster kinetics of hydration advancement degree $\alpha_{th}$ from the IST (Figure 5b), leading to a larger content of C-(N)-A-S-H (Table 8) and a larger reduction in porosity. Finally, MS is characterized by the longest setting to reach PT, which is related to the lower pH of its pore solution (Figure 1). This leads to a slow formation of C-(N)-A-S-H (Table 8).

The main key points of this study on the hydration process at early age are summarized as follows:

- During the 3 first days, the sodium sulphate activator proves to be the most effective activator for BFS hydration. It is followed by the potassium sulphate, while dihydrated calcium sulphate and magnesium sulphate lead to slower hydration. This difference of behavior is due to their lower pH and their cation nature.
- The thermal activation can accelerate the hydration process, particularly for BFS activated with sodium, potassium and dihydrated calcium sulphate as shown by their activation energy coefficients.
- The good correlations between the heat flow evolution and two characteristic times of setting, IST and PT, lead to propose a time of final setting equal to the “Plateau time” determined with the ultrasonic waves for the alkali activation of BFS. This approach is also in good agreement with the evolution of the hydration advancement degree *α_th_* and the formation kinetics of C-(N)-A-S-H- and M-S-H-type gels.
- The coupling of the studied parameters (heat flow, hydration advancement degree, kinetics of hydrates formation, setting) indicate an important delay of the hydration process of MS. This behavior is investigated more in depth with thermodynamic modeling (Section 3.6.1).

### 3.4. Formation of the Hydration Products Beyond 3 Days

**Figure 10:**
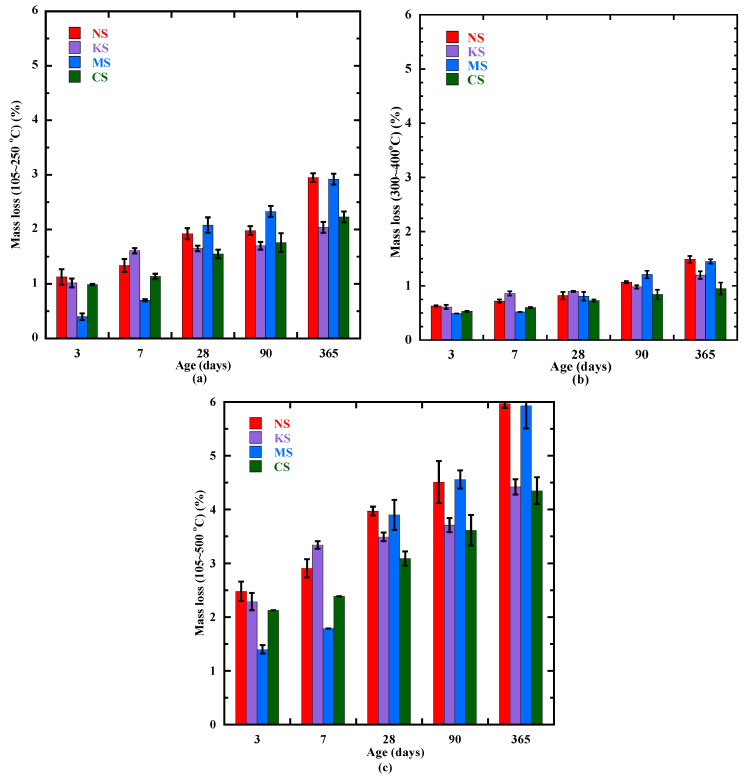
Evolution of mass loss related to the decomposition of (**a**) C-(N)-A-S-H- and M-S-H-type gels, (**b**) hydrotalcite, (**c**) chemical-bound water from 3 to 365 days.

Beyond 3 days, the formation of the hydration products is determined using three different experimental techniques: TGA, XRD and SEM+EDS. The formation kinetics of C-(N)-A-S-H- and M-S-H-type gels, hydrotalcite and chemical-bound water is presented in Figure 10. The mass loss measured from 105 °C to 250 °C associated with the content of C-(N)-A-S-H- and M-S-H-type gels (Figure 10a) is increasing until 1 year for all the studied mortars due to continuous hydration advancement [54], but with different kinetics. A similar content of these gels (about 1% of mass loss) at 3 days is observed for NS, KS and CS, whereas the content is twice as low for MS. Until 7 days, the C-(N)-A-S-H content progresses faster for KS with a mass loss equal to 1.6%, but it slows down beyond the first week. At 7 days, MS always presents a lower content of C-(N)-A-S-H- and M-S-H-type gels and its evolution significantly accelerates beyond the first week. During the first month, the evolution of hydrotalcite (Figure 10b) and chemical-bound water (Figure 10c) contents of all the studied mixtures presents a kinetics quite similar to that observed for the content of C-(N)-A-S-H- and M-S-H-type gels. So, the activators of sodium and potassium sulphate favor the formation of hydrates during the first week as shown by chemical-bound water evolution (Figure 10c).

**Figure 11:**
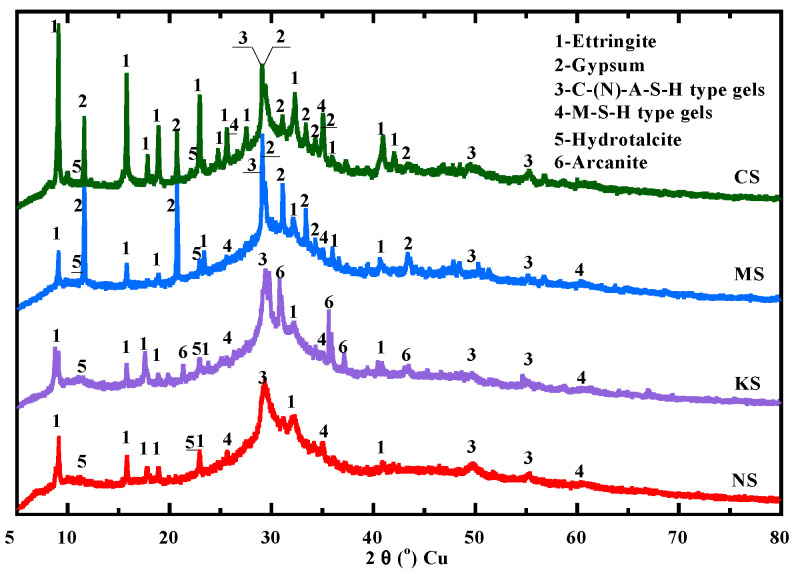
Hydration products identified with XRD for all studied mixtures at 28 days.

As the mass loss related to hydrotalcite is low (inferior to 1%), the main hydration products during the first month are C-(N)-A-S-H- and M-S-H-type gels. At 28 days, the mass loss related to these gels is equal to 2% for MS and NS, 1.6% for KS and 1.5% for CS. These products are also clearly detected on XRD measurements (Figure 11) with a peak mainly identified for C-(N)-A-S-H [50,92] at about 29.2°, and for M-S-H gels at about 35° [93,94], respectively. The peak at 11.3° identified for hydrotalcite [95] (ICDD No. 41-1428) is insignificant in comparison to other products as shown by the XRD patterns (Figure 11). Due to a high content of sulphate ions into the pore solution, ettringite [95,96] (ICDD No. 41-1451) is the main second product. Gypsum [95,97] (ICDD No.33-311) is also observed for CS and MS. Finally, KS also presents another crystalline phase: arcanite K_2_SO_4_ [95,98] (ICDD No.5-613) possibly due to an excess of potassium ions from the alkali-sulphate activation.

**Figure 12:**
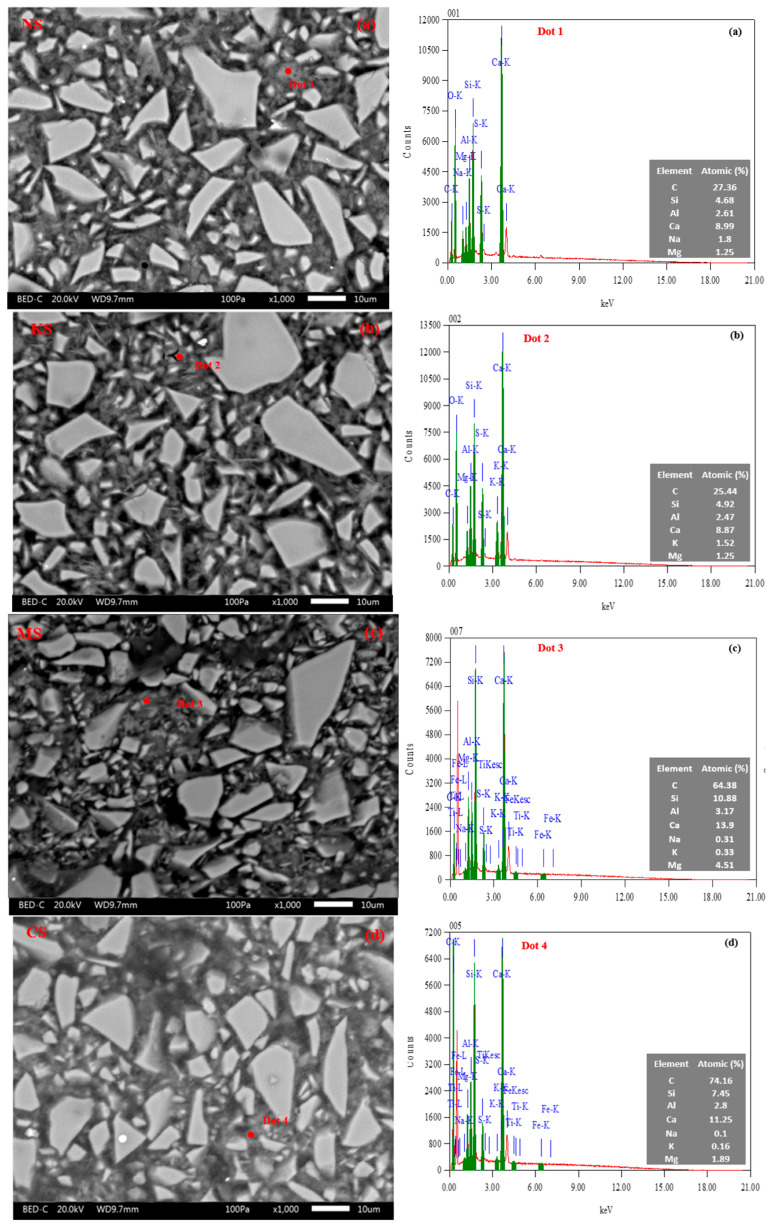
A typical SEM-BSE image and an EDS result indicated by the red dot on the SEM image for NS (**a**), KS (**b**), MS (**c**) and CS (**d**) at 28 days.

**Figure 13:**
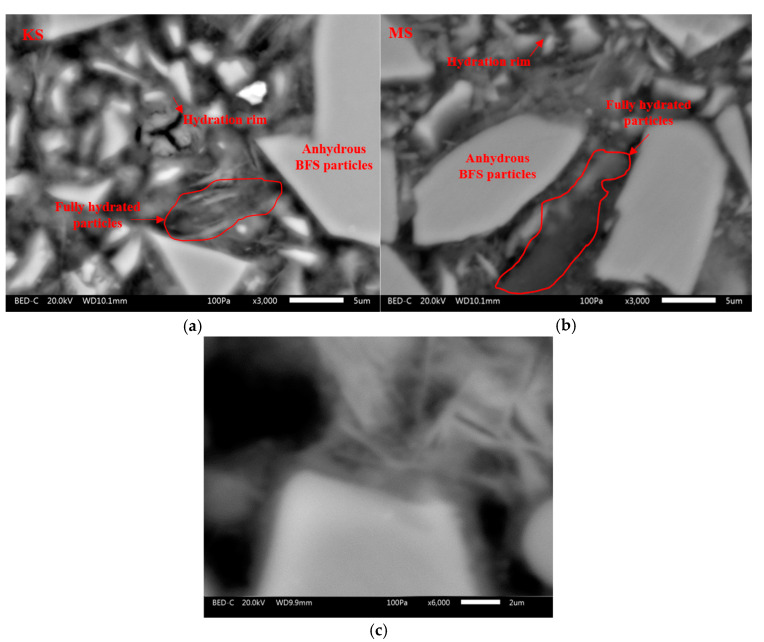
BSE images with a higher magnification for KS (**a**), MS (**b**) and NS (**c**) at 28 days.

The hydrated pastes at 28 days are also observed with SEM-BSE coupled with EDS to determine the effect of the activator type on the nature of C-(N)-A-S-H gel and the distribution of the hydration products into the matrix. In these pictures (Figure 12), the anhydrous BFS particles are characterized by a white irregular shape surrounded by a hydrated rim with a thin layer inferior to 1 μm as shown on Figure 13a,b. Between the anhydrous BFS particles, the gel presents a foil-like morphology as shown in Figure 13c. This morphology was already observed for C-A-S-H-type gels of alkali-activated BFS [50,99,100,101] and hydrated CEM Ⅲ-type cement at 28 days [102]. It is difficult to identify the portlandite, resulting from the hydration of Portland cement.

The results of EDS analysis (atomic contents and elemental ratios) of C-(N)-A-S-H/M-S-H-type gels at 28 days are given in Table 9 and Table 10 for all the studied mixtures. The Ca/Si ratio varies from 1.3 to 1.6, a value quite similar to that observed for Portland and blended blast-furnace slag and alkali-activated slag (AAS) cement (Table 10) [50,59,100,101,102]. However, the chemical nature of the activator directly affects the concentration of some ions into the hydrated phase. Indeed, the contents of Na, K and Mg are the highest for NS (1.9%), KS (1.8%) and MS (3.7%), respectively (Table 9). They modify the chemical composition of the C-(N)-A-S-H-type gel in replacing some Ca ions by new ions (Na, K, e.g.). The MS mixture shows the highest Mg/Al ratio equal to 1.5, which is found in M-S-H-type gels identified by the XRD pattern in Figure 11. The concentration of aluminum ions is also more important in the hydrated phases as shown by the more important Al/Si ratio (0.3~0.5) and the lower Ca/(Si+Al) ratio (~1.1) for all the studied mixtures in comparison to the values observed for PC (0.1 and 1.3, respectively) [50]. It is strongly related to the chemical composition of BFS (Table 2). This confirms the presence of C-(N)-A-S-H/M-S-H-type gels.

Beyond 28 days, the formation of C-(N)-A-S-H/M-S-H-type gels (Figure 10) evolves rapidly for NS and MS, as well as for the hydrotalcite and chemical-bound water. Indeed, a similar value of mass loss characterizes NS and MS at 365 days for C-(N)-A-S-H/M-S-H-type gels (2.9%), hydrotalcite (1.5%) and chemical-bound water (4.5%) (Figure 10). These results indicate that the sodium sulphate activator also leads to a better activation of BFS in the long term, resulting in a larger content of C-(N)-A-S-H from 3 to 365 days. This is possibly related to two main factors: (1) the highest pH of the pore solution (Figure 1) and (2) the cation interaction with C-A-S-H-type gel [46,51,92]. Indeed, the pH increases significantly for NS from 12.1 at 3 days to 12.8 at 365 days compared to KS (12.3–12.7) (Figure 1). It is possibly due to the chemical reaction of Na_2_SO_4_ with Ca^2+^ and the production of NaOH [103]. A higher pH and a higher alkalinity of the pore solution leads to a more important dissolution of BFS particles and favorizing the development of C-(A)-S-H-type gel. The behavior of MS in the long term differs from that at early age by the formation of a more important content of C-(N)-A-S-H/M-S-H-type gels. This is due to the fact that the pH increases (Figure 1) and the Mg^2+^ content in the pore solution continues to decrease from 7 to 365 days (Figure 2). It is possible that more available Mg^2+^ are bound in the M-S-H-type gel (Table 10 & [99,101]). The second factor is related to the effect of cations. It has been found [25,46,92] that Na^+^ is easier to incorporate and to absorb in the C-A-S-H-type gel in comparison to K^+^ in the alkali activation of BFS systems, thus forming more C-(N)-A-S-H. This behavior was also observed for some alkali-activated metakaolin-slag systems and geopolymer systems [25,104].

### 3.5. Hydration of BFS

To determine clearly the effect of the activator type on the hydration process, the degree of hydration is a useful tool. It also offers an indication on the material performances as a high degree of hydration leads to good mechanical strength [8], but it is a necessary input parameter for thermodynamic modeling.

**Figure 14:**
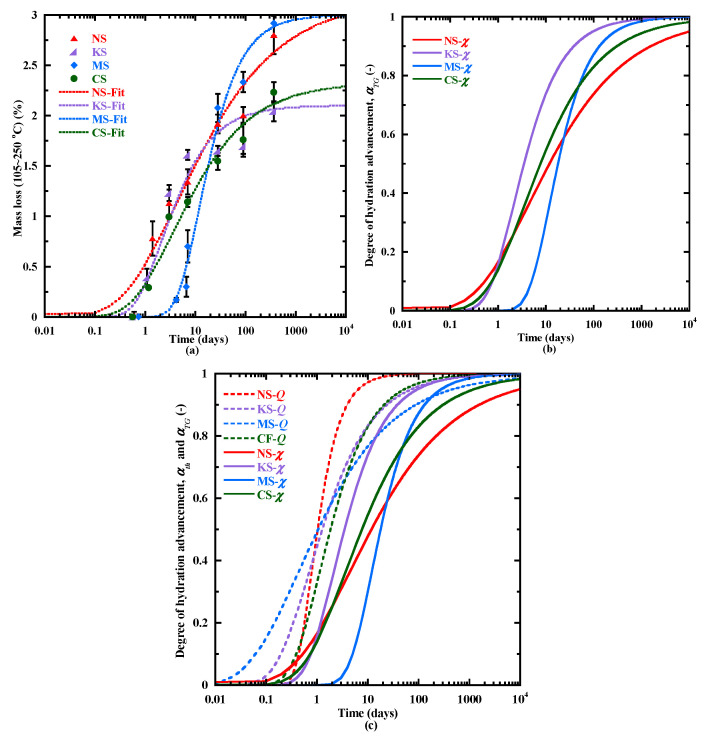
Mass loss related to C-(N)-A-S-H/M-S-H-type gels measured with TGA and the fitting curves with the CPM model (**a**), the degree of hydration advancement $\alpha_{TG}$ as a function of time (**b**) and comparison of hydration degree advancement $\alpha_{th}$ and $\alpha_{TG}$ (**c**).

In Section 3.3.1, a hydration advancement degree $\alpha_{th}$ (Figure 5b) is proposed basing on the cumulative heat evolution determined with the isothermal calorimetry (Figure 5a). This approach offers us only an indication of the hydration kinetics during the first days. It is directly due to the low sensibility of the heat flow measurement beyond several days. To overcome this, another hydration advancement degree ($\alpha_{TG}$) is proposed based on the mass loss evolution of the C-(N)-A-S-H/M-S-H type gels (Figure 10) as these gels are the main hydrates from the setting to 1 year. The degree of hydration advancement $\alpha_{TG}$ is calculated with CPM (Equations (8) nd (9)). The numerical parameters of the model (Equation (8)) obtained by fitting it on the curves of mass loss related to C-(N)-A-S-H/M-S-H-type gels (Figure 14a) are given in the Table 11. The evolution of $\alpha_{TG}$ is presented in Figure 14b. At 1 day, $\alpha_{TG}$ is quite similar for NS, KS and CS (about 0.2). From 1 to 10 days, the kinetics of $\alpha_{TG}$ is faster for KS. Beyond 10 days, MS presents a stronger increase in $\alpha_{TG}$, gradually exceeding the values of $\alpha_{TG}$ for NS and CS. At 28 days, $\alpha_{TG}$ is close for MS (0.69) and CS (0.72), but lower for NS (0.65) and significantly higher for KS (0.9). It confirms the highest potential of hydration of NS in the long term. Beyond 100 days, KS first reaches its final value of $\alpha_{TG}$, followed by MS. On the contrary, the hydration advancement degree of CS and NS is increasing beyond several years, confirming their higher hydration capacity in the long term, particularly with the sodium sulphate activator.

The evolution of both degrees of hydration advancement $\alpha_{th}$ and $\alpha_{TG}\;$ are shown in Figure 14c. The first approach ($\alpha_{th}$) offers more information on the hydration kinetics at early age, particularly during the setting, whereas the second ($\alpha_{TG}$) shows a higher sensibility in the long term. The results show that the sodium sulphate allows accelerating the hydration at early age but also maintaining a hydration process in the long term. On the other hand, magnesium sulphate leads to a hydration reduction and an early hydration stop. However, these approaches only allow comparing the kinetics of the hydration process. To determine evolution of hydration degree $\alpha_{BFS}$, a new methodology is proposed with the calculation of the percentage of anhydrous BFS particles at 28 days and the evolution of $\alpha_{TG}$ (Figure 14b). The percentage of anhydrous BFS particles is determined using image analysis, as this method allows for accurate segmentation of anhydrous particles using various filters and grey level thresholding. Compared to selective dissolution, image analysis is more reliable because selective dissolution often leads to excessive dissolution of hydrates or partial dissolution of anhydrous BFS particles when different acids or complexing agents are used [86].

**Figure 15:**
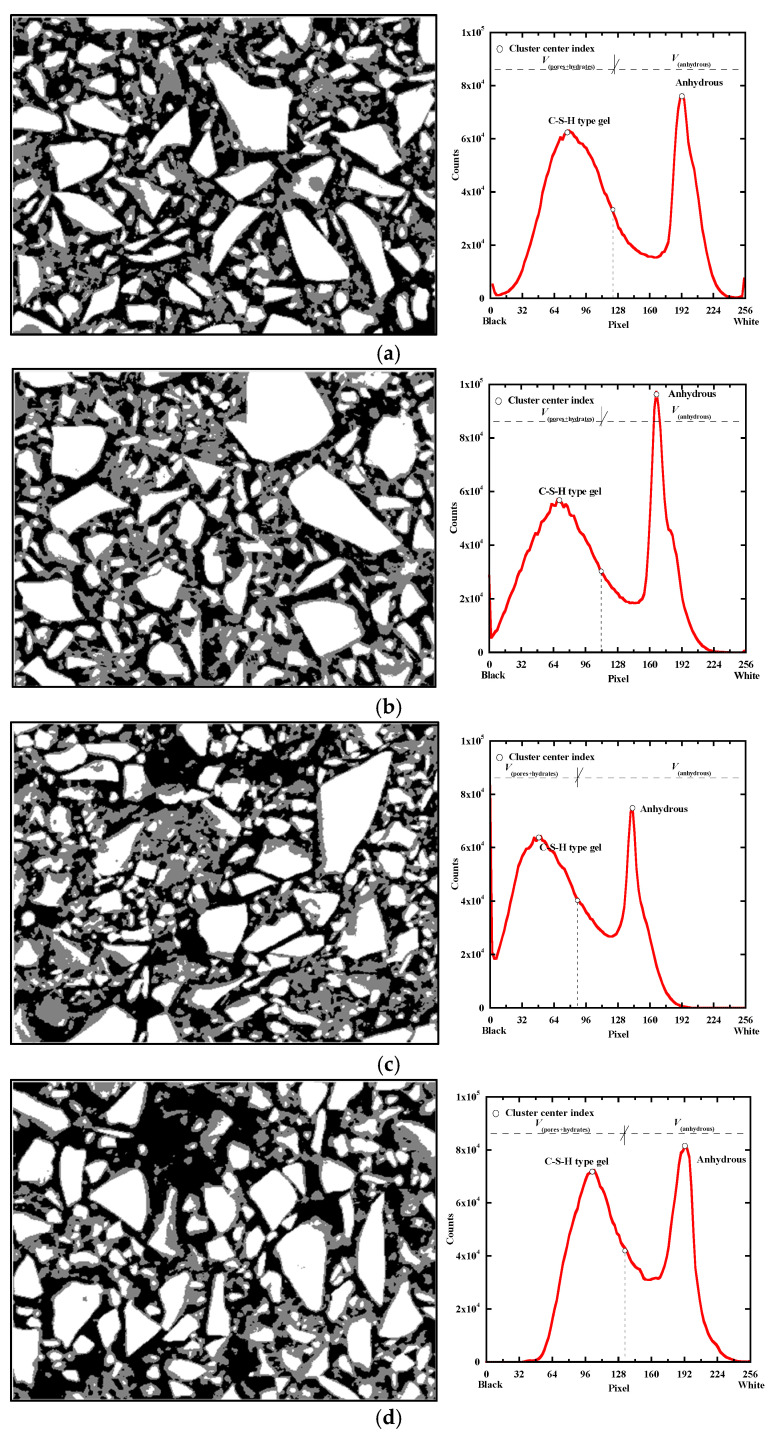
Segmentation of BSE images for NS (**a**), KS (**b**), MS (**c**), CS (**d**) and their thresholding based on the K-means clustering method.

The anhydrous BFS volume fraction (${Vf}_{anhyd-BFS}$) at 28 days is determined thanks to a K-means clustering method used to segment the BSE-SEM images [54,59]. This technique allows displaying clearly the main anhydrous phases (white areas in Figure 15 on the left) in a picture. After segmentation, the data representing anhydrous BFS are concentrated in the range of the highest pixel values of the histogram of the grey levels, as illustrated in Figure 15 (on the right). It allows the quantification of unreacted blast-furnace slag particles and of the hydration degree of BFS ($\alpha_{BFS})$ at 28 days with Equations (12) and (13) using three micrographs for each mixture.

(12) $${Vf}_{anhyd-BFS}(t=0)(\%)=\frac{V_{BFS}(t=0)}{V_{BFS}\left(t=0\right)+{V_{PC}\left(t=0\right)+V}_{water}\left(t=0\right)+V_{air}\left(t=0\right)}$$

(13) $$\alpha_{BFS}(t=28)=\frac{{Vf}_{anhyd-BFS}\left(t=0\right)-{Vf}_{anhyd-BFS}\left(t=28\right)}{{Vf}_{anhyd-BFS}\left(t=0\right)}$$

where $\alpha_{BFS}$ is the hydration degree of BFS, *Vf_anhyd-BFS_* is the volume fraction of anhydrous BFS particles. *V_BFS_* (*t* = 0), V_PC_ (*t* = 0) and *V_water_* (*t* = 0) are the initial volume fraction of anhydrous BFS particles, Portland cement and water (m^3^), respectively. *V_air_* (*t* = 0) is the initial content of air (Table 3).

**Figure 16:**
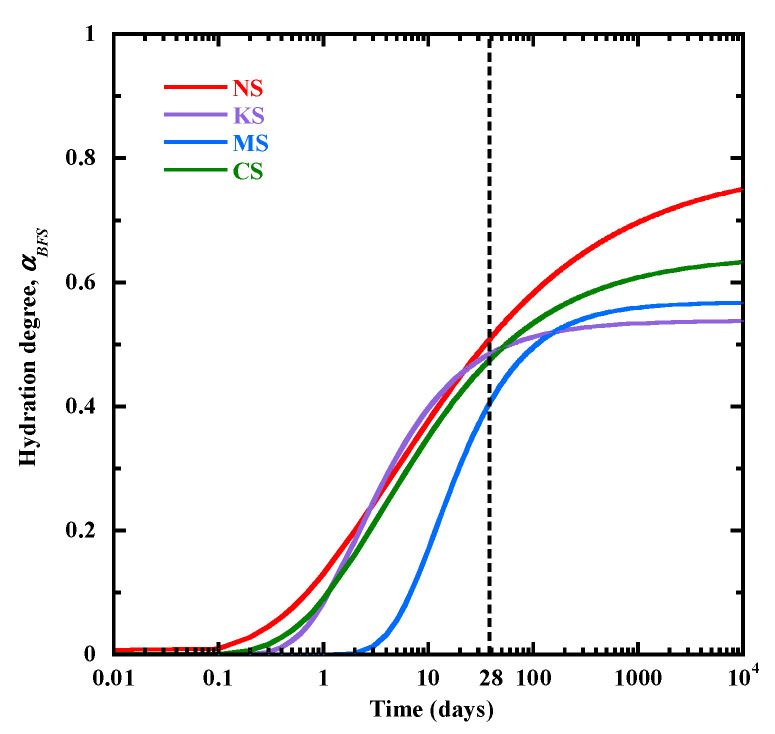
The evolution of BFS hydration degree, $\alpha_{BFS}.$

Table 12 presents the anhydrous BFS volume fraction (*Vf*_anhyd-*BFS*_ (*t* = 28)) obtained from the K-mean clustering segmentation method and the initial volume fraction of BFS particles just after mixing (*Vf*_anhyd-*BFS*_ (*t* = 0)). The anhydrous BFS volume fraction at 28 days is quite similar for NS, KS and CS (about 34%), but it is higher for MS (42%). Based on these values and Equation (12), the BFS hydration degree $\alpha_{BFS}\;$ is determined at 28 days and it is quite close for NS, KS and CS (around 0.47), and lower for MS (0.36). The evolution of the hydration degree of BFS ($\alpha_{BFS}$) is calculated with Equation (14) and presented in Figure 16 for all the studied mixtures. $\alpha_{BFS}\;$increases faster at very early age for NS and its evolution is quite similar for NS, KS and CS from 2 days to 20 days. Until 28 days, a larger hydration degree of BFS is observed for NS and KS. It can be attributed to their higher pH levels (Figure 1). MS presents a notable increase in hydration degree from 3 to 28 days. It is correlated with a continuous reduction in Mg^2+^ contents in the pore solution (Figure 2c), leading to an increase in the BFS dissolution during this period and favoring the formation of M-S-H-type gels (Figure 10). Finally, the evolution rate of $\alpha_{BFS}\;$decreases first for KS, followed by MS and CS. Their final values of $\alpha_{BFS}$ are equal to 54%, 57% and 64%, respectively. But NS shows the slowest deceleration and the highest final value of $\alpha_{BFS}$ (79%), confirming its strongest hydration capability.

(14) $$\alpha_{BFS}(t)=\alpha_{TG}\left(t\right)\frac{\alpha_{BFS}(t=28)}{\alpha_{TG}\left(t=28\right)}$$

### 3.6. Applications for the Hydration Degree of BFS

Finally, two applications are proposed for the hydration degree of BFS: a thermodynamic modeling of the hydration process of the studied alkali-activated pastes as a function of this parameter as well as compressive strength evolution.

#### 3.6.1. Hydration Modeling

**Figure 17:**
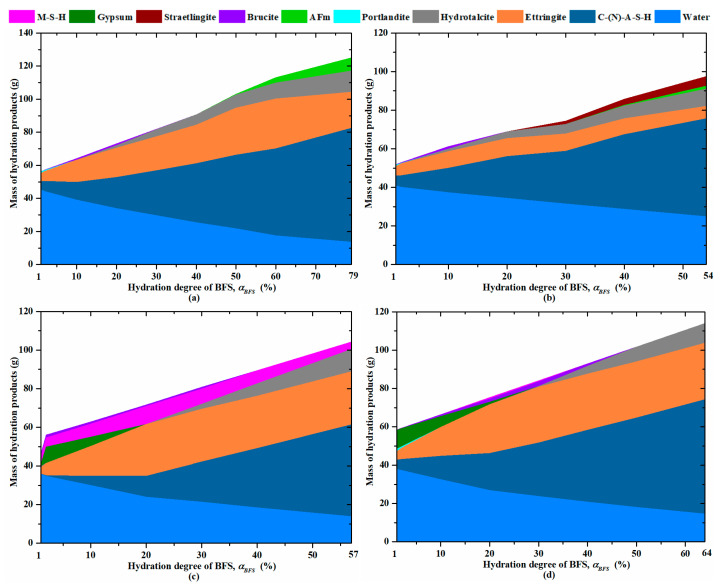
Thermodynamic modeling of BFS-activated mortars: (**a**) NS (**b**) KS (**c**) MS (**d**) CS.

Using the thermodynamic modeling GEMS and the CEMDATA18 database, the evolution of the hydration products as a function of the hydration degree of slag ($\alpha_{BFS})$ is simulated for the four studied alkali-activated pastes. This thermodynamic modeling allows enhancing the understanding of their hydration process. The results are presented in Figure 17. The percentages of hydrates given hereafter are calculated as a function of the total content of hydrates at the correspondent hydration degree of BFS.

It appears that the ettringite content is significant at early age for all the studied mixtures (48% for NS, 32% for KS, 30% for MS and 26% for CS at $\alpha_{BFS}=0.02$). It decreases progressively for KS and NS as a part of ettringite is replaced by AFm when $\alpha_{BFS}$ becomes superior to 0.4 and 0.5, respectively. This reduction is amplified for KS due to the formation of straetlingite in the long term, a siliceous AFm-type phase stable in the C-A-S-H system [66,105]. At the final hydration degree of BFS, the ettringite content reaches a value equal to 20% for NS, 8% for KS, 30% for MS and CS. The second main product for NS, KS and CS is C-(N)-A-S-H in agreement with the TGA and SEM observations (Figure 10 and Figure 12). At $\alpha_{BFS}=0.02$, its content is equal to 47%, 32% and 26%, respectively. For MS, its content is very low (1%), but another type of gel is present in a large content: M-S-H (22% at $\alpha_{BFS}=0.02$). The content of C-(N)-A-S-H continuously increases with the hydration process, in agreement with the results presented in Figure 14a. Thus, it is correct to choose this parameter to monitor the advancement of the hydration reaction as in Figure 14b. At the final value of the hydration degree of BFS, the content of C-(N)-A-S-H is equal to about 62%, 64%, 60% and 53% for NS, KS, CS and MS, respectively. As a function of the activator type, other hydrates are also present at early age in a large content: gypsum for CS and MS (about 40% at $\alpha_{BFS}=0.02$) and arcanite (33% at $\alpha_{BFS}=0.02$). These hydrates are also observed on the XRD graphs (Figure 11). Their content decreases with the hydration process, and the total content of gypsum is consumed for $\alpha_{BFS}$ superior to 0.1 and 0.2 for MS and CS, respectively. The M-S-H gel observed for MS also decreases with the hydration process. It is progressively replaced by C-(N)-A-S-H-type gel and hydrotalcite. The transformation of M-S-H into hydrotalcite is possible thanks to the availability of the aluminium anions from the BFS dissolution and a high pH of the pore solution (Figure 1) [106,107].

At very early age ($\alpha_{BFS}<0.1)$, a small content of portlandite (<10%) is also produced for NS, KS and CS due to the hydration of PC, but it decreases progressively and it is rapidly totally consumed. No portlandite is produced for MS. That explains the significant delay observed for its hydration process at early age (Figure 3) and for its setting (Figure 8). Due to the high content of OH^−^ as shown by the pH values (Figure 1) and the presence of Mg^2+^ (Figure 2c) in the pore solution, brucite (Mg(OH)_2_) is rapidly formed for all the studied pastes at early age [105]. Its content is more important for MS (7% at $\alpha_{BFS}=0.02$, e.g.,) due to its highest content of Mg^2+^ in the pore solution (Figure 2c). With the advancement of the hydration process, its content also decreases until totally disappearing beyond $\alpha_{BFS}$ equal to 0.1 for KS, 0.3 for NS and MS and 0.4 for CS. Indeed, brucite is progressively replaced by hydrotalcite. As hydrotalcite presents a more complex structure characterized by Layered Double Hydroxides (LDHs) and as its formation needs the substitution of Mg^2+^ by Al^3+^ in the LDH [105,106,108], it appears later and its content increases continuously. At the final value of the hydration degree of BFS, its content is equal to about 13% for MS, 11% for NS and KS and 10% for CS.

#### 3.6.2. Evolution of the Compressive Strength

**Figure 18:**
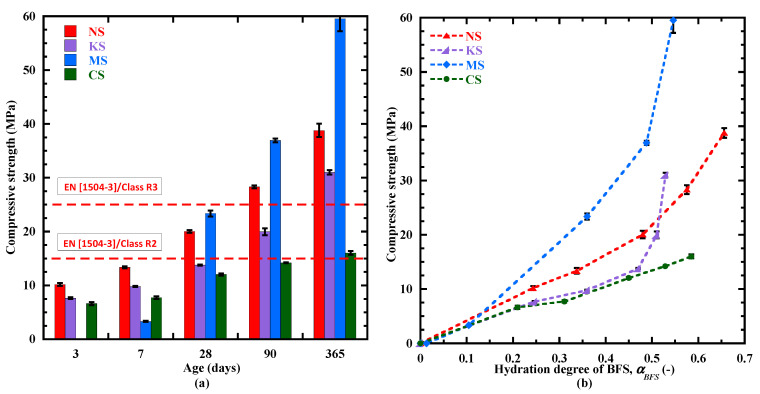
Evolution of compressive strength from 3 to 365 days (**a**) and as function of hydration degree of BFS (**b**).

Finally, the evolution of compressive strength, one of the main material properties, is correlated with the hydration degree of BFS ($\alpha_{BFS})$ in Figure 18b. It is also presented as a function of time in Figure 18a. During the first week, the highest compressive strength is observed for NS (10 MPa at 3 days and 13 MPa at 7 days) due to its larger content of C-(N)-A-S-H-type gel and ettringite (Figure 17a). These products are beneficial for compressive strength in reducing the pore volume and in improving the chemical bonds between hydrates. But no strength can be determined for MS, related to its delayed setting, earlier than the final setting of −213 h, and its compressive strength at 7 days. The compressive strength of MS is low (3 MPa) due to its low value of $\alpha_{BFS}\;\left(0.1\right)$ (Figure 16 and Figure 18b) and its low content of hydrates, particularly in C-(N)-A-S-H-type gel (Figure 17c). So, the early-age strength at 7 days is more indicatable, as it shows a comparable result after setting for all the studied sulphate-activated materials. At 28 days, the compressive strength is equal to 24 MPa for MS, 20 MPa for NS, 14 MPa for KS and 12 MPa for CS. NS and MS have the potential to satisfy the strength requirement of repaired mortars for structural (R3) and non-structural (R2) applications (EN 1504-3 [109]). Beyond 28 days, this ranking of the mortars is always valid, but the increase rate of strength is more important for MS. For thee same value of $\alpha_{BFS}$ (Figure 18b), the classification of strength from $\alpha_{BFS}=0$.1 to 0.5 is the following: MS > NS > KS ≥ CS. The superior strength of NS and MS can be explained by their larger contents of C-(N)-A-S-H-type gel, ettringite and hydrotalcite. Moreover, MS contains a significative content of M-S-H, and this hydrate is beneficial to the mechanical strength [50,110,111], and the coexistence with C-S-H-type gel may amplify this effect, leading to finer porosity and reduced pore volume [94,111].

## 4. Conclusions

In this research study, a large experimental program investigates the effect of the cation nature on the hydration mechanisms and the compressive strength of alkali-activated materials with a high content of sulphate ions. For these materials, a novel approach for determining the evolution of the hydration degree of blast-furnace slag is also proposed to correlate hydrate evolution with compressive strength. The following conclusions can be drawn:

1. Three experimental techniques (calorimetry, TGA, ultrasonic measurement) applied on the sulphate-activated mortars indicate that sodium sulphate is the most effective activator for the BFS hydration at early age (≤3 days). This is evidenced by its fast hydration kinetics and setting due to the fast formation of C-(N)-A-S-H-type gel and ettringite. For the potassium sulphate and dihydrated calcium sulphate activators, their hydration kinetics is slower due to slower C-(N)-A-S-H-type gel formation and a lower initial pH of the pore solution, leading to a lower rate of hydration advancement evolution. For these three activators, the thermal activation is significant, as shown by their higher coefficient of activation energy. A different behavior characterizes the mortar activated with magnesium sulphate: a delay in the hydration process and the largest setting times due to the absence of portlandite and the delay in the formation of C-(N)-A-S-H-type gel.
2. Beyond 28 days, the kinetics of hydration products is the most significant for mortars activated with sodium and magnesium sulphate. In the long term, they present a larger content of hydrates, such as C-(N)-A-S-H-type gels, ettringite and hydrotalcite. Moreover, magnesium sulphate contributes to the formation of a large content of M-S-H-type gel due to an increase in the pH of the pore solution and a continuous consumption of Mg^2+^. All these hydrates provide a denser matrix, leading to a satisfying compressive strength for standard repaired mortars.
3. The new approach proposed to predict the hydration degree of blast-furnace slag as a function of time is based on the evolution to C-(N)-A-S-H/M-S-H-type gels measured with TGA and the content of unhydrated particles of blast-furnace slag quantified by the SEM technique. The relevance of this approach is confirmed by the results from thermodynamic modeling, confirming a continuous increase in C-(N)-A-S-H for the mortars activated with alkali sulphates.

## Figures and Tables

**Table 1:** Main characteristics of the studied activators at 20 °C.

Chemical Activators	Na_2_SO_4_	K_2_SO_4_	MgSO_4_	CaSO_4_.2H_2_O
Density (g/cm^3^)	2.70	2.66	2.65	2.32
Molar mass (g/mol)	142	174	120	172
Solubility (g/100 mL)	19.5	11.0	35.0	2.0
pH in distilled water	10.2	10.4	9.5	9.2
Purity (%)	99	-	98	98

**Table 2:** Main characteristics of the studied activators at 20 °C.

Chemical Composition	SiO_2_	CaO	Fe_2_O_3_	Al_2_O_3_	MgO	T_2_O	SO_3_	Na_2_O	Cl^−^	K_2_O	Fineness (cm^2^/g)
BFS	35.1	42.1	0.4	11.1	7.0	0.8	0.1	0.2	0.03	0.4	4200
PC	20.8	65.9	2.2	5.4	1.1	-	3.4	0.2	-	0.3	4682

**Table 3:** Mortar compositions and properties in fresh state.

Compositions	NS	KS	MS	CS
PC (kg/m^3^)	29	29	29	26
Activator (kg/m^3^)	46	56	39	60
BFS (kg/m^3^)	502	492	509	433
Water (kg/m^3^)	243	242	242	247
Sand (kg/m^3^)	1444	1444	1444	1444
Binder (kg/m^3^)	577	577	577	519
PC/B	0.05	0.05	0.05	0.05
A/B	0.08	0.10	0.07	0.11
BFS/B	0.87	0.85	0.88	0.84
Water/B	0.42	0.42	0.42	0.50
Paste volume (L/m^3^)	443	443	443	443
SO_4_^2−^ concentration (mol/L)	1.4	1.4	1.4	1.4
Activator state ^a^	TD	PD	TD	SP
Average slump (mm)	26.0	13.0	6.3	6.1
Average air content (%)	4.9	4.5	4.5	4.4

^a^: TD: Total Dissolution—PD: Partial Dissolution—SP: Solid Phase.

**Table 4:** The characteristic times of the hydration kinetics and the ultimate cumulative heat at 20 °C ($Q_{\left(\infty ,\;20\right)}$).

Characteristic Times of the Hydration Kinetics	NS	KS	MS	CS
Duration of the dormant period (h)	5.4	7.0	31.0	2.5
The peak before the dormant period named “*the 2nd peak*”(h)	-	3.1	3.3	7.6
The peak during the acceleration period named “*the 3rd peak*” (h)	21.2	28.6	199	32.1
The beginning of the acceleration period (b.a.p) (h)	9.4	13.6	63.0	11.1
Duration from the end of the dormant period to *the 3rd peak* (h)	11.8	15.0	136	21
$Q_{\infty ,20}$ (J/g_binder_)	161	110	84	122

**Table 5:** The average mass loss for different temperature intervals for the beginning of the acceleration period (b.a.p.), the second and third peaks of heat flow curves, and at 72 h.

Mass Loss (%)	NS			KS				MS				CS			
	Beginning of the Acceleration Period (9 h)	3rd Peak (21 h)	72 h	2nd Peak (3 h)	Beginning of the Acceleration Period (14 h)	3rd Peak (29 h)	72 h	2nd Peak (3 h)	Beginning of the Acceleration Period (63 h)	72 h	3rd Peak (199 h)	2nd Peak (7 h)	Beginning of the Acceleration Period (11 h)	3rd Peak (32 h)	72 h
C-(N)-A-S-H and M-S-H-type gel (105–250 °C)	0	0.2	1.13	0	0	0.4	1.02	0	0.3	0.4	0.5	0	0	0.3	0.99
Hydrotalcite and brucite (300–450 °C)	0.1	0.3	0.60	0	0.06	0.3	0.59	0.01	0.4	0.48	0.5	0.03	0.05	0.3	0.53
Portlandite (400–500 °C)	0.1	0.2	0.36	0.02	0.05	0.2	0.32	0.01	0.3	0.33	0.35	0.03	0.06	0.2	0.33
Chemical-bound water (105–500 °C)	0.3	0.9	2.43	0.02	0.1	1.0	2.29	0.02	1.0	1.3	1.4	0.05	0.13	0.8	2.13

**Table 6:** The evolution rate of C-(N)-A-S-H- and M-S-H-type gels during the acceleration and deceleration periods, the evolution rate of *α_th_* during the acceleration period and pH values.

Mixtures	Evolution Rate of Mass Loss from 105 to 250 °C (‰/h)		$\mathrm{Evolution}\;\mathrm{Rate}\;\mathrm{of}\;\alpha_{th}$ (-) Acceleration Period	pH After Mixing	pH at 3 Days
	Acceleration Period	Deceleration Period			
NS	4.7	0.7	3.8	12.77	12.07
KS	3.0	1.5	3.3	12.65	12.26
MS	0.02	-	0.6	11.91	11.50
CS	2.0	1.6	2.0	11.93	12.25

**Table 7:** Thermal and mechanical parameters used to calculate *E_a_*, the activation energy coefficients obtained with the superposition (*E_a,SM_*) and velocity (*E_a,VM_*) methods as well as some characteristic times.

Parameters	NS			KS			MS			CS		
	10 °C	20 °C	30 °C	10 °C	20 °C	30 °C	10 °C	20 °C	30 °C	10 °C	20 °C	30 °C
$Q_{\infty ,\;10}$$,\;Q_{\infty ,\;20}$$,\;Q_{\infty ,\;30}$ (J/g_binder_)	159	161	137	143	110	133	85	83	60	144	122	119
${\bar{Q}}_{\left(\infty \right)}$ (J/g_binder_)	152			129			76			128		
*Q_inf_* (J/g_binder_)	24			21			22			21		
*Q_sup_* (J/g_binder_)	91			77			42			76		
*R*_c,28_ (MPa)	20			14			23			12		
*E*_a,*SM*_ (kJ/mol)	38			40			33			39		
*E*_a*,VM*_ (kJ/mol)	46			45			37			52		
Maximal value of *E*_a,*VM*_ (kJ/mol)	111			76			83			89		
Time corresponding to the beginning of the 1st Stage (h)	12			7			8			13		
Time (h) and *α_th_* (-) corresponding to the maximal *E_a_*	15–0.26			11–0.27			12–0.38			17–0.24		

**Table 8:** The characteristic times of setting (IST, FST and PT) at 20 °C and their corresponding velocity and mass loss from 105 °C to 250 °C ($∆m_{105-250{}^{\circ}C}$) determined with TGA for all the studied mortars.

Mixtures	NS			KS			MS			CS		
	Time (h)	Velocity (m/s)	$∆m_{105-250{}^{\circ}C}$ (%)	Time (h)	Velocity (m/s)	$∆m_{105-250{}^{\circ}C}$ (%)	Time (h)	Velocity (m/s)	$∆m_{105-250{}^{\circ}C}$ (%)	Time (h)	Velocity (m/s)	$∆m_{105-250{}^{\circ}C}$ (%)
Initial Setting Time (IST)	15.5	2167	0.00 ± 0.0	14.0	2480	0.00 ± 0.0	17.0	1940	0.00 ± 0.0	13.4	1691	0.00 ± 0.0
Interval from IST to FST	17.5	630	0.85	12.0	400	0.40	140.0	715	0.26	20.1	640	0.40
Final Setting Time (FST)	33.0	2797	0.85 ± 0.08	26.0	2879	0.40 ± 0.06	157.0	2655	0.26 ± 0.07	33.5	2331	0.40 ± 0.05
Interval from FST to PT	6.7	358	0.10	14.0	291	0.18	56.0	461	0.56	13.1	554	0.2
Plateau Time (PT)	39.7	3155	0.90 ± 0.05	40.0	3170	0.58 ± 0.05	213.0	3116	0.82 ± 0.1	46.6	2885	0.60 ± 0.02

**Table 9:** Average atomic contents of the hydrated phase for all the studied mixtures.

Element (Atom%) ^a^	Ca	Si	Al	Na	K	Mg
NS	8.3 ± 0.5	5.7 ± 0.8	2.2 ± 0.3	1.9 ± 0.1	-	1.7 ± 0.4
KS	8.6 ± 0.2	5.4 ± 0.4	2.4 ± 0.2	-	1.8 ± 0.2	1.4 ± 0.2
MS	12.7 ± 1.8	8.8 ± 1.0	2.6 ± 0.3	0.3 ± 0.1	0.3 ± 0.0	3.7 ± 0.6
CS	12.6 ± 0.5	8.3 ± 3.7	2.0 ± 0.2	0.1 ± 0.1	0.2 ± 0.2	1.3 ± 0.2

^a^ average of 3 or 6 measurements.

**Table 10:** The main elemental ratios of the hydrated phases for all the studied mixtures.

Element Ratio (-)	Ca/Si	Al/Si	Ca/(Si+Al)	Al/(Na or K or Mg or Ca)	Mg/Al
NS	1.5 ± 0.3	0.4 ± 0.1	1.1 ± 0.1	0.20 ± 0.0	0.8 ± 0.3
KS	1.6 ± 0.2	0.5 ± 0.1	1.1 ± 0.0	0.18 ± 0.0	0.7 ± 0.1
MS	1.5 ± 0.3	0.3 ± 0.0	1.1 ± 0.1	0.15 ± 0.1	1.5 ± 0.1
CS	1.5 ± 0.4	0.3 ± 0.1	1.2 ± 0.1	0.16 ± 0.0	0.6 ± 0.1
AAS [50,101,102]	1.2~2.0	0.0~0.2	0.6	-	1.0~1.8
PC [99]	1.5~2.0	0.0~0.1	1.3	-	-
BFS/PC [58,102]	1.0~2.0	-		-	1.7~2.7

**Table 11:** Parameters for CPM determined with the evolution of the mass loss related to C-(N)-A-S-H/M-S-H-type gels.

Mixtures	NS	KS	MS	CS
$\chi_{\left(\infty \right)}$ (%)	3.1	2.1	3	2.3
$\tau_{TG}$ (h)	4.6	2.2	12.2	3.8
$a_{TG}$ (-)	0.4	0.8	0.9	0.5

**Table 12:** Main parameters for determining the BFS hydration degree ($\alpha_{BFS}$).

Mixtures	NS	KS	MS	CS
*V*_anhyd-*BFS*_ (*t* = 28) (%)	34.0 ± 1.4	34.8 ± 2.0	42.0 ± 2.9	33.4 ± 0.7
*V*_pores + hydrates_ (*t* = 28) (%)	66.0 ± 3.0	65.2 ± 3.2	58.0 ± 4.9	65.6 ± 1.8
*Vf*_anhyd-*BFS*_ (*t* = 0) (%)	65.4	65.9	66.2	62.3
$\alpha_{BFS}$ (*t* = 28)	0.48	0.47	0.36	0.45
$\alpha_{TG}$ (*t* = 28)	0.61	0.87	0.63	0.70
$\alpha_{BFS}\;\left(t=\infty \right)$	0.79	0.54	0.57	0.64

## Data Availability

The raw data supporting the conclusions of this article will be made available by Lei Li on request.

## Appendix A. Temperature Effect on the Hydration Process

**Table A1:** The characteristic times of the hydration kinetics and the ultimate cumulative heat at 10, 20 and 30 °C.

Characteristics Times	NS			KS			MS			CS		
	10 °C	20 °C	30 °C	10 °C	20 °C	30 °C	10 °C	20 °C	30 °C	10 °C	20 °C	30 °C
Duration of the dormant period (h)	20.8	5.4	2.4	9.5	7.0	2.0	80.0	31.0	22.0	9.8	2.5	1.8
*the 2nd peak* (h)	46.3	21.2	10.6	2.8	3.1	1.6	9.2	3.3	1.6	13.2	7.6	11.5
*the 3rd peak* (h)	-	-	-	55.5	28.6	13.5	328	199	130	54.5	32.1	15.3
The beginning of the acceleration period (b.a.p) (h)	25.6	9.4	4.3	17.5	13.6	4.5	130	63.0	42.0	23.0	11.1	2.9
Duration from the end of the dormant period to *the 3rd peak* (h)	20.7	11.8	6.3	38	15	9	198	136	88	31.5	21	8.6
$Q_{\infty ,\;10}$$,\;Q_{\infty ,\;20}$$,\;Q_{\infty ,\;30}$ (J/g_binder_)	159	161	137	143	110	133	85	83	60	144	122	119
${\bar{Q}}_{\left(\infty \right)}$ (J/g_binder_)	152			129			76			128		
